# Association of the pathomics-collagen signature with lymph node metastasis in colorectal cancer: a retrospective multicenter study

**DOI:** 10.1186/s12967-024-04851-2

**Published:** 2024-01-25

**Authors:** Wei Jiang, Huaiming Wang, Xiaoyu Dong, Yandong Zhao, Chenyan Long, Dexin Chen, Botao Yan, Jiaxin Cheng, Zexi Lin, Shuangmu Zhuo, Hui Wang, Jun Yan

**Affiliations:** 1 Department of General Surgery, Guangdong Provincial Key Laboratory of Precision Medicine for Gastrointestinal Tumor, Nanfang Hospital, The First School of Clinical Medicine, Southern Medical University, Guangzhou, Guangdong 510515 People’s Republic of China; 2 School of Science, Jimei University, Xiamen, Fujian 361021 People’s Republic of China; 3 Department of General Surgery (Colorectal Surgery), The Sixth Affiliated Hospital, Sun Yat-Sen University, Guangzhou, Guangdong 510655 People’s Republic of China; 4 Guangdong Provincial Key Laboratory of Colorectal and Pelvic Floor Diseases, The Sixth Affiliated Hospital, Sun Yat-Sen University, Guangzhou, Guangdong 510655 People’s Republic of China; 5 Biomedical Innovation Center, The Sixth Affiliated Hospital, Sun Yat-Sen University, Guangzhou, Guangdong 510655 People’s Republic of China; 6 Department of Pathology, The Sixth Affiliated Hospital, Sun Yat-Sen University, Guangzhou, Guangdong 510655 People’s Republic of China; 7 Division of Colorectal and Anal Surgery, Department of Gastrointestinal Surgery, Guangxi Medical University Cancer Hospital, Nanning, 530000 People’s Republic of China; 8 Department of Gastrointestinal Surgery, Shenzhen People’s Hospital, Second Clinical Medical College of Jinan University, First Affiliated Hospital of Southern University of Science and Technology, Shenzhen, Guangdong 518020 People’s Republic of China

**Keywords:** Colorectal cancer, Lymph node metastasis, Pathomics, Collagen features

## Abstract

**Background:**

Lymph node metastasis (LNM) is a prognostic biomarker and affects therapeutic selection in colorectal cancer (CRC). Current evaluation methods are not adequate for estimating LNM in CRC. H&E images contain much pathological information, and collagen also affects the biological behavior of tumor cells. Hence, the objective of the study is to investigate whether a fully quantitative pathomics-collagen signature (PCS) in the tumor microenvironment can be used to predict LNM.

**Methods:**

Patients with histologically confirmed stage I-III CRC who underwent radical surgery were included in the training cohort (n = 329), the internal validation cohort (n = 329), and the external validation cohort (n = 315). Fully quantitative pathomics features and collagen features were extracted from digital H&E images and multiphoton images of specimens, respectively. LASSO regression was utilized to develop the PCS. Then, a PCS-nomogram was constructed incorporating the PCS and clinicopathological predictors for estimating LNM in the training cohort. The performance of the PCS-nomogram was evaluated via calibration, discrimination, and clinical usefulness. Furthermore, the PCS-nomogram was tested in internal and external validation cohorts.

**Results:**

By LASSO regression, the PCS was developed based on 11 pathomics and 9 collagen features. A significant association was found between the PCS and LNM in the three cohorts (*P* < 0.001). Then, the PCS-nomogram based on PCS, preoperative CEA level, lymphadenectasis on CT, venous emboli and/or lymphatic invasion and/or perineural invasion (VELIPI), and pT stage achieved AUROCs of 0.939, 0.895, and 0.893 in the three cohorts. The calibration curves identified good agreement between the nomogram-predicted and actual outcomes. Decision curve analysis indicated that the PCS-nomogram was clinically useful. Moreover, the PCS was still an independent predictor of LNM at station Nos. 1, 2, and 3. The PCS nomogram displayed AUROCs of 0.849–0.939 for the training cohort, 0.837–0.902 for the internal validation cohort, and 0.851–0.895 for the external validation cohorts in the three nodal stations.

**Conclusions:**

This study proposed that PCS integrating pathomics and collagen features was significantly associated with LNM, and the PCS-nomogram has the potential to be a useful tool for predicting individual LNM in CRC patients.

**Supplementary Information:**

The online version contains supplementary material available at 10.1186/s12967-024-04851-2.

## Background

The incidence of colorectal cancer (CRC) has been increasing over the last few decades, ranking among the top three cancers in terms of prevalence and mortality [1–5]. Lymph node metastasis (LNM) is the most important metastatic model of CRC and results in a poor prognosis [6, 7]. LNM also influences treatment strategy determination, such as local treatment, including endoscopic resection and local excision, in patients with early-stage colon cancer due to the low probability of LNM [8, 9]. Moreover, the likelihood of LNM is a critical indicator of whether patients with rectal cancer receive neoadjuvant treatment [8, 9]. Thus, the accurate estimation of LNM in CRC patients is crucial for tailored treatment. However, the diagnostic accuracy of LNM based on medical imaging data in patients with CRC is currently not satisfactory. The sensitivity ranges only from 55 to 73%, and the specificity ranges between 74 and 78% from CT images in CRC patients [10–12].

With the improvement of technology in the field of medical science, microscopes have gradually been replaced by digitalization. Whole hematoxylin and eosin (H&E)-stained slides of the specimen are scanned and stored as a digital pathological image [13]. These images are widely recognized and contain a wealth of pathological information, including tumor cells and the tumor microenvironment (TME) [14]. Furthermore, this information could be quantified by the digital pathology analysis technique named "pathomics" [15, 16]. Recently, the term “pathomics” has attracted increased attention. Pathomics is an interdisciplinary field that integrates pathology with high-throughput image analysis, computational modelling, and machine learning methods. The objective of this approach is to extract valuable information from digital pathology images and subsequently analyse this information to improve disease diagnosis and prognosis prediction [13, 15–17].

Collagen in the TME is significantly correlated with the biological behavior of tumor cells, such as adhesion, invasion, and metastasis [18, 19]. However, traditional pathological images cannot be used to visualize collagen structure in the TME. Multiphoton imaging (MPI) is a nonlinear optics-based microscopic imaging technique that includes 2-photon excitation fluorescence (TPEF) from cells and second harmonic generation (SHG) from collagen [20, 21]. Notably, MPI is a powerful tool for investigating the structural changes in collagen during the occurrence and development of various diseases [22], such as neoadjuvant treatment response in breast cancer [23], ovarian cancer invasive metastasis [24] and prostate cancer recurrence [25]. Furthermore, quantifiable collagen features can be extracted from multiphoton images and indicated as valuable biomarkers for diagnosis and prognosis prediction [26–28]. Therefore, collagen can be used as a complement to pathomics.

Our hypothesis is that integrating pathomics from digital H&E images and collagen features from multiphoton images is a feasible approach to thoroughly elucidate the relationship between the tumor with its microenvironment and LNM. To enhance predictive accuracy, it has been accepted that incorporating multiple biomarkers into a single signature is preferable to analyzing each biomarker individually [29, 30]. Least absolute shrinkage and selection operator (LASSO) regression is an effective algorithm for selecting and shrinking high-dimensional parameters and is commonly used for model construction. Hence, this study aims to propose a quantitative pathomics-collagen signature (PCS) based on pathomics features and collagen structure via LASSO regression to synthetically elucidate the association between the tumor with its microenvironment and LNM and then construct a PCS-nomogram that incorporates the PCS and clinicopathological predictors for estimating the probability of LNM in CRC patients.

## Methods

### Patients and specimens

Ethical approval was obtained for this retrospective analysis, and the informed consent requirement was waived (NFEC-2022-222 and 2022ZSLYEC-267). The study was conducted following the guidelines of the Declaration of Helsinki and the Standards for Reporting Diagnostic Accuracy (STARD) statement criteria.

The inclusion criteria were as follows: (1) patients ≥ 18 years old; (2) patients diagnosed with CRC according to pathological examination; (3) patients who underwent radical surgery with harvested lymph nodes ≥ 12; and (4) patients with available specimen slides. The exclusion criteria were as follows: (1) synchronous malignant neoplasms; (2) previous neoadjuvant treatment; (3) positive margin; and (4) distant metastasis. A total of 658 consecutive patients were recruited from Nanfang Hospital between January 2014 and December 2016. These patients were randomly assigned in a 1:1 ratio to training and internal validation cohorts. An independent external validation cohort included 315 consecutive patients with the same criteria from the Sixth Affiliated Hospital, Sun Yat-sen University, between January 2014 and December 2014 (Additional file 1: Fig. S1). The formalin-fixed paraffin-embedded specimens of all patients were used.

Baseline clinicopathological characteristics included age, sex, primary tumor location, preoperative CEA level, preoperative CA19-9 level, lymphadenectasis on CT, tumor differentiation, venous emboli and/or lymphatic invasion, and/or perineural invasion (VELIPI), tumor size, and pathological T stage.

### Digital pathological image acquisition, selection of regions of interest, and extraction of pathomics features

The digital pathological images of H&E-stained slides were inspected using an Aperio ScanScope Scanner system (Leica Biosystems) with a 20 × objective. These images were saved in.SVS format and then managed using Aperio ImageScope software (version 12.3.3). Two independent pathologists who were blinded to the nodal status selected the most representative area of tumor invasion for each image. When the two pathologists disagreed, the final decision was made by the director of the pathology department. Five regions of interest (ROIs) with a field of view of 500 × 500 μm were randomly selected from the chosen area and saved as TIF format files. Then, a total of 114 pathomics features were extracted from the files using CellProfiler software (version 4.1.3), which is a free and open-source platform for the quantitative analysis of biological images [31, 32]. The pathomics features are summarized in Additional file 1: Table S1. The average pathomics feature value of the five files was used for subsequent statistical analysis. Details of the pathomics feature extraction are provided in the Additional file 1: Supplementary Methods.

### Multiphoton image acquisition and extraction of collagen features

Five ROIs on the H&E-stained slide, which were selected for the extraction of pathomics features, were subjected to MPI with a 20 × objective. Subsequently, the multiphoton image was compared to the digital H&E image for histologic assessment. A total of 142 collagen features were extracted from the multiphoton image by MATLAB 2018b (MathWorks) (Additional file 1: Table S2) [27]. The above steps were performed by an optical expert who was unaware of the nodal status. Details of the MPI system and collagen extraction can be found in the Additional file 1: Additional Methods.

### Feature selection and PCS construction

LASSO regression, which is a suitable algorithm for analysing high-dimensional data, utilizes an L1 penalty to shrink some regression coefficients to exactly zero, which could effectively shrink the regression coefficients and select predictive features to avoid overfitting and covariance. The penalty parameter λ, also referred to as the tuning constant, dictates the penalty's strength in regulating the number of parameters entering the model. The optimal value of λ was determined by tenfold cross-validation with 1—standard error criterion in the training cohort [33, 34]. The calculation formula of the PCS was acquired. Then, the PCS for each patient was directly calculated based on the calculation formula. More information about the LASSO regression can be found in the Additional file 1: Additional Methods.

### Development and evaluation of the PCS-nomogram

The PCS and clinicopathological characteristics were included in univariate analysis to analyze their relationship with LNM, and variables with *P* < 0.10 were included in multivariable analysis. A backward stepwise selection method with Akaike’s information criterion as the stopping rule was used to select the independent predictors of LNM [35]. The prediction model was constructed based on multivariable logistic analysis in the training cohort and presented as a PCS-nomogram. The multicollinearity of the PCS-nomogram was estimated via the variance inflation factor (VIF) [36].

The performance of the PCS-nomogram was assessed via the area under the receiver operating characteristic curve (AUROC) and calibration curve. Then, the PCS-nomogram was applied in the internal and external validation cohorts. The ROC curves of the models were compared using the DeLong method.

### Clinical application value of the PCS-nomogram

To evaluate the clinical application value of the PCS-nomogram. A clinicopathological characteristic-based model (i.e., the traditional model) was used for comparison with the PCS-nomogram. Decision curve analysis (DCA) was used to identify the clinical usefulness [37, 38]. The specificity, sensitivity, accuracy, negative predictive value (NPV), and positive predictive value (PPV) were measured according to the maximum Youden index value of the ROC curve of the two models. In addition, the net reclassification improvement (NRI) and integrated discrimination improvement (IDI) were calculated to compare the performance outcomes of the PCS-nomogram and traditional model [39, 40]. Details of DCA, NRI, and IDI are provided in the Additional file 1: Additional Methods.

### Statistical analysis

Categorical variables were compared using the chi-square test or Fisher’s exact test. Continuous variables were compared by Student’s t test or the Mann‒Whitney *U* test. The odds ratio (OR) and 95% confidence interval (CI) of the predictors were calculated using multivariable logistic regression. Survival curves were generated by using the Kaplan–Meier method and compared by log-rank tests. Univariate and multivariable analyses with Cox proportional hazards regression determined the hazard ratio (HR) of predictors for disease-free survival (DFS) and overall survival (OS). All statistical analyses were performed with SPSS version 22.0 software and R version 4.0.3. All *P* values were two-sided, and statistical significance was defined as *P* < 0.05.

## Results

### Clinicopathological characteristics

The clinicopathological characteristics of the training cohort (n = 329), the internal validation cohort (n = 329) and the external validation cohort (n = 315) are listed in Table 1. The median ages (interquartile range, IQR) were 60 (51.0, 66.0) years, 59 (51.0, 67.0) years, and 58 (50.0, 66.0) years in each cohort. The median (IQR) number of lymph nodes harvested was 25.0 (18.0, 30.0), 25.0 (17.0, 30.0), and 24 (18.0, 31.0) in the three cohorts, respectively. The rates of LNM were 44.7% (147/329), 45.6% (150/329), and 49.0% (155/315) in the three cohorts. There were no significant differences among the three cohorts in LNM prevalence (*P* = 0.479). The clinicopathological characteristics were similar among the three cohorts, which justified their use as training and validation cohorts (Table 1).

**Table 1:** Characteristics of the patients in the training, internal validation and external validation cohorts

Characteristic	Training cohort (n = 329)	Internal validation cohort (n = 329)	External validation cohort (n = 315)	*P*
Age, median (IQR)	60.0 (51.0, 66.0)	59.0 (51.0, 67.0)	58 (50.0, 66.0)	0.401
Sex, No. (%)				0.807
Male	189 (57.4)	196 (59.6)	188 (59.7)	
Female	140 (42.6)	133 (40.4)	127 (40.3)	
Primary tumor location, No. (%)				0.434
Left-sided	221 (67.2)	213 (64.7)	219 (69.5)	
Right-sided	108 (32.8)	116 (35.3)	96 (30.5)	
Preoperative CEA level, No. (%)				0.196
Normal	200 (60.80	197 (59.9)	171 (54.3)	
Elevated	129 (39.2)	132 (40.1)	144 (45.7)	
Preoperative CA19-9 level, No. (%)				0.394
Normal	251 (76.3)	247 (75.1)	226 (71.7)	
Elevated	78 (23.7)	82 (24.9)	89 (28.3)	
Lymphadenectasis on CT, No. (%)				0.648
< 10 mm	153 (46.5)	147 (44.7)	135 (42.9)	
≥ 10 mm	176 (53.5)	182 (55.3)	180 (57.1)	
Tumor differentiation, No. (%)				0.105
Well or moderately	264 (80.2)	266 (80.9)	235 (74.6)	
Poorly or undifferentiated	65 (19.8)	63 (19.1)	80 (25.4)	
VELIPI, No. (%)				0.661
No	192 (58.4)	189 (57.4)	173 (57.9)	
Yes	137 (41.6)	140 (42.6)	142 (45.1)	
Tumor size, cm, No. (%)				0.622
< 4	144 (43.8)	136 (41.3)	142 (45.1)	
≥ 4	185 (56.20	193 (58.7)	173 (54.9)	
pT stage, No. (%)				0.137
pTis-T2	71 (21.6)	69 (21.0)	45 (14.3)	
pT3	145 (44.1)	145 (44.1)	147 (46.7)	
pT4	113 (34.3)	115 (35.0)	123 (39.0)	
Lymph node metastasis				0.479
Yes	147 (44.7)	150 (46.6)	155 (49.0)	
No	182 (55.3)	179 (53.4)	160 (51.0)	
PCS, median (IQR)	− 0.251 (− 0.505, 0.386)	− 0.244 (− 0.484, 0.210)	− 0.249 (− 0.395, 0.038)	0.118

Values in parentheses are percentages unless indicated otherwise

*VELIPI* venous emboli and/or lymphatic invasion and/or perineural invasion, *PCS* pathomics-collagen signature, *IQR* interquartile range

### Construction of the PCS

**Fig. 1:**
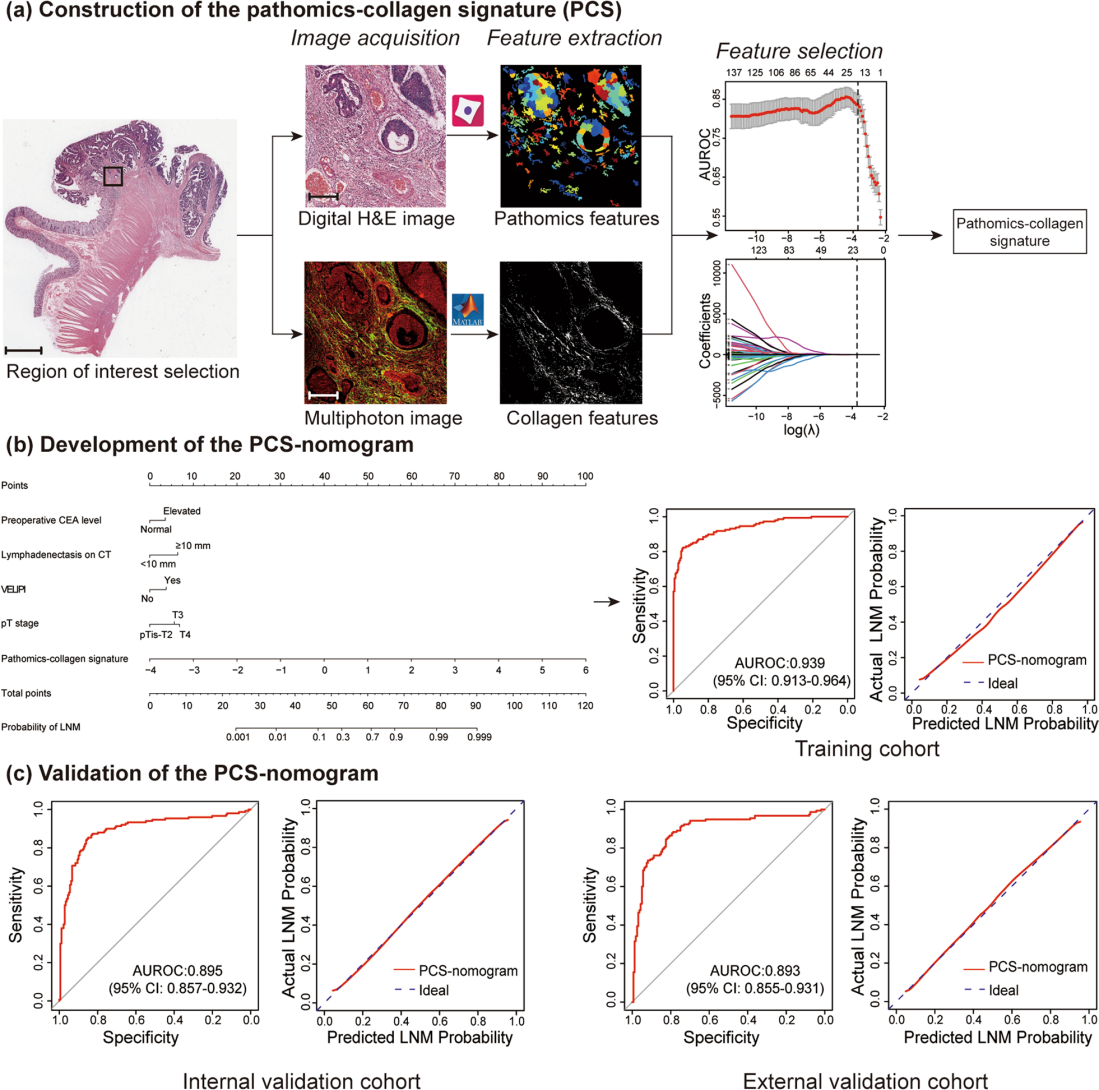
Workflow of this study. **a** Selection of the region of interest on a digital H&E image. The selected region of interest was used to extract pathomics features by CellProfiler software. The region of interest was subjected to multiphoton imaging. Then, collagen features were extracted from the multiphoton image by MATLAB 2018b. LASSO regression was used to select the most predictive parameters to construct the PCS. **b** The PCS-nomogram was developed based on the PCS and four clinicopathological predictors to predict LNM in the training cohort. **c** The PCS-nomogram was verified in the internal and external validation cohorts. Scale bars: 1000 μm and 200 μm. *PCS* pathomics-collagen signature, *LASSO* least absolute shrinkage and selection operator, *VELIPI* venous emboli and/or lymphatic invasion and/or perineural invasion, *LNM* lymph node metastasis, *AUROC* area under the receiver operating characteristic curve

**Fig. 2:**
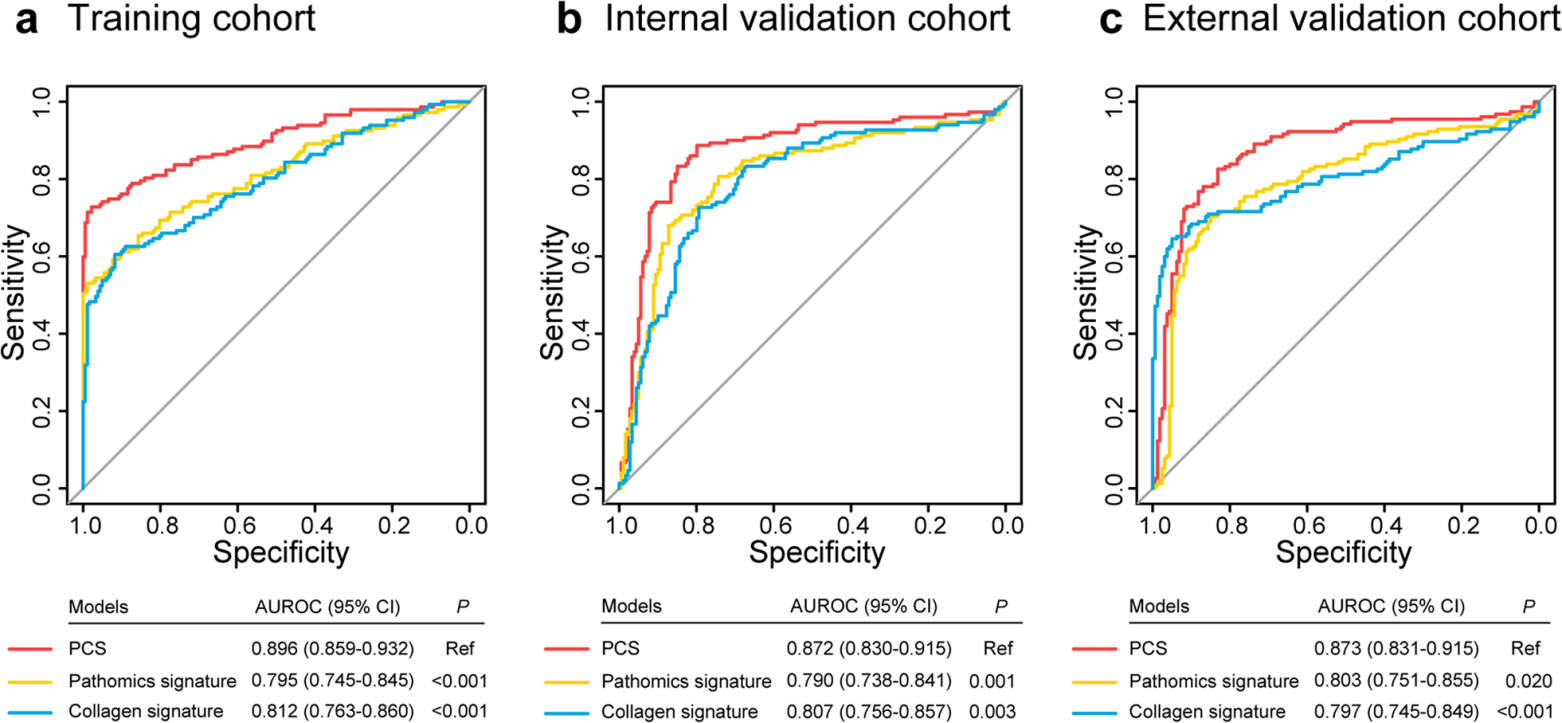
ROC curves of the pathomics-collagen signature versus single-modality prediction models in the three cohorts. ROC curves of the PCS, pathomics signature, and collagen signature in the training cohort (**a**) and internal (**b**) and external (**c**) validation cohorts. *ROC* receiver operating characteristic, *LNM* lymph node metastasis, *CRC* colorectal cancer

The flowchart of this study is shown in Fig. 1. Of the pathomics features and collagen features, the twenty most predictive features via LASSO regression were used to build the PCS, which included 11 pathomics features and 9 collagen features (Additional file 1: Fig. S2). The calculation formula of PCS is presented in the Additional file 1: Supplementary Results. The PCS yielded AUROCs of 0.896 (95% CI, 0.859–0.932), 0.872 (95% CI, 0.830–0.915), and 0.873 (95% CI, 0.831–0.915) in the training, internal validation and external validation cohorts, respectively. Furthermore, when performing stratified analysis, we found a significant association between PCS and LNM (Additional file 1: Table S3). Compared with PCS, the pathomics signature model (Additional file 1: Fig. S3a, b) and the collagen signature model (Additional file 1: Fig. S3c, d) had significantly lower AUROCs ranging from 0.790 to 0.803. The PCS indicated better predictive performance for estimating LNM than the single-modality prediction models in the three cohorts (*P* < 0.05) (Fig. 2).

### Development and evaluation of the PCS-nomogram

**Fig. 3:**
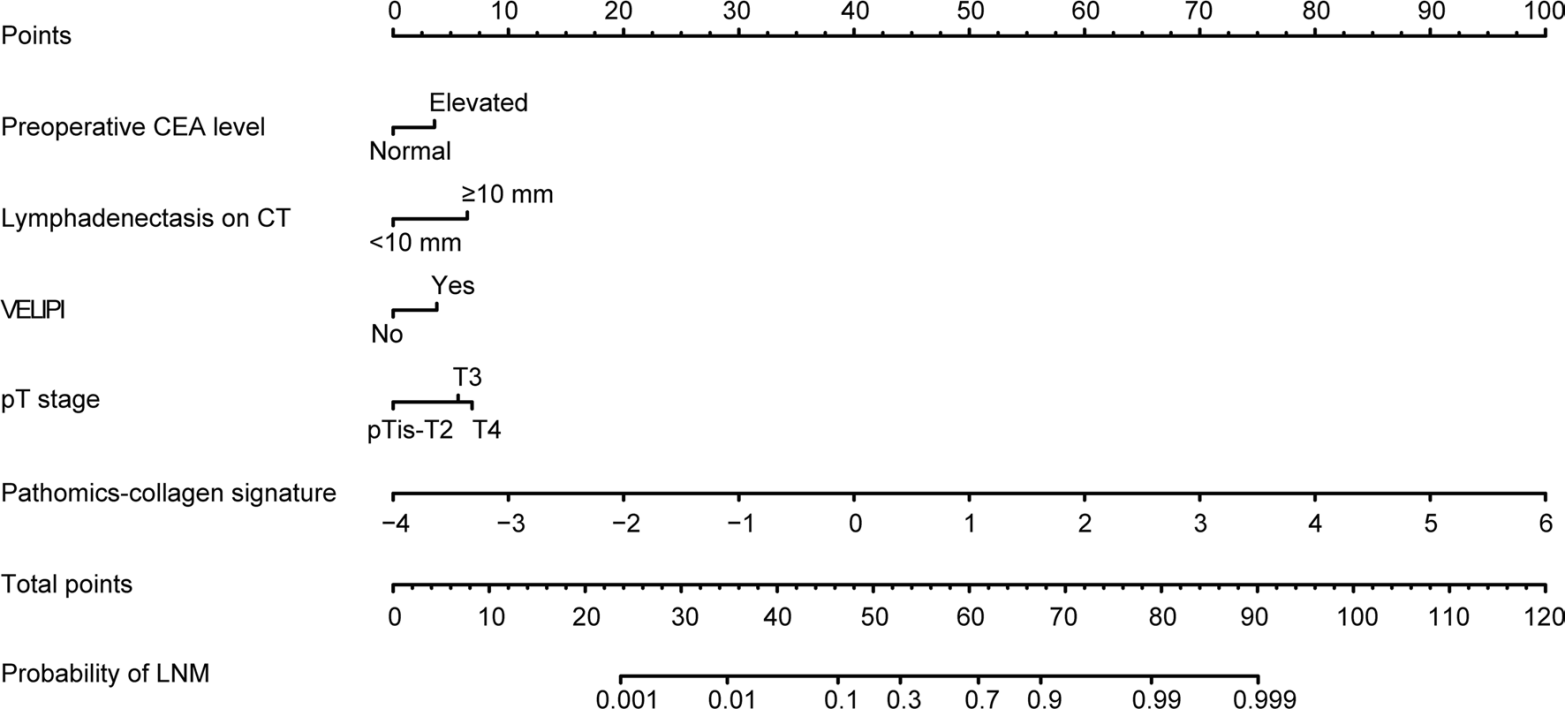
PCS-nomogram to predict LNM in patients with CRC. The PCS-nomogram was developed based on the preoperative CEA level, lymphadenectasis on CT, VELIPI, pT stage, and PCS to predict the probability of LNM in patients with CRC. *CRC* colorectal cancer, *VELIPI* venous emboli and/or lymphatic invasion and/or perineural invasion, *LNM* lymph node metastasis

Univariate analysis demonstrated that preoperative CEA level, lymphadenectasis on CT, tumor differentiation, VELIPI, pT stage, and PCS were potential predictors of LNM in the training cohort (all *P* < 0.10) (Table 2). Multivariable analysis showed that preoperative CEA level, lymphadenectasis on CT, VELIPI, pT stage, and PCS were independent predictors of LNM (all *P* < 0.05) (Table 2). The PCS indicated significantly better discrimination than the other predictors (Additional file 1: Fig. S4). Then, a prediction model comprising the above five independent predictors was constructed and proposed as the PCS-nomogram (Fig. 3). The VIF of each predictor was less than 10; thus, there was no multicollinearity among these predictors (Additional file 1: Fig. S5).

**Table 2:** Univariate and multivariable analyses of predictors of LNM in the training cohort

Variables	Univariate analysis		Multivariable analysis	
	OR (95% CI)	*P*	OR (95% CI)	*P*
Age	0.985 (0.968, 1.003)	0.104		
Sex				
Male	Ref.			
Female	1.023 (0.659, 1.587)	0.920		
Primary tumor location				
Left-sided	Ref.			
Right-sided	1.042 (0.656, 1.655)	0.860		
Preoperative CEA level				
Normal	Ref.		Ref.	
Elevated	2.105 (1.342, 3.302)	0.001	2.109 (1.010, 4.405)	0.047
Preoperative CA19-9 level				
Normal	Ref.			
Elevated	1.417 (0.851, 2.358)	0.180		
Lymphadenectasis on CT				
< 10 mm	Ref.		Ref.	
≥ 10 mm	4.381 (2.736, 7.015)	< 0.001	3.816 (1.834, 7.943)	< 0.001
Tumor differentiation				
Well or moderately	Ref.			
Poorly or undifferentiated	1.583 (0.918, 2.732)	0.099	NA	NA
VELIPI				
No	Ref.		Ref.	
Yes	3.074 (1.949, 4.848)	< 0.001	2.198 (1.067, 4.572)	0.033
Tumor size, cm				
< 4	Ref.			
≥ 4	1.182 (0.762, 1.833)	0.455		
pT stage				
pTis-T2	Ref.		Ref.	
pT3	2.262 (1.208, 4.237)	0.011	3.239 (1.156, 9.080)	0.025
pT4	4.135 (2.153, 7.941)	< 0.001	4.162 (1.396, 12.411)	0.011
PCS	7.943 (4.521, 13.955)	< 0.001	8.014 (4.266, 12.140)	< 0.001

*OR* odds ratio, *CI* confidence interval, *VELIPI* venous emboli and/or lymphatic invasion and/or perineural invasion, *NA* not available, *Ref* reference, *PCS* pathomics-collagen signature

**Fig. 4:**
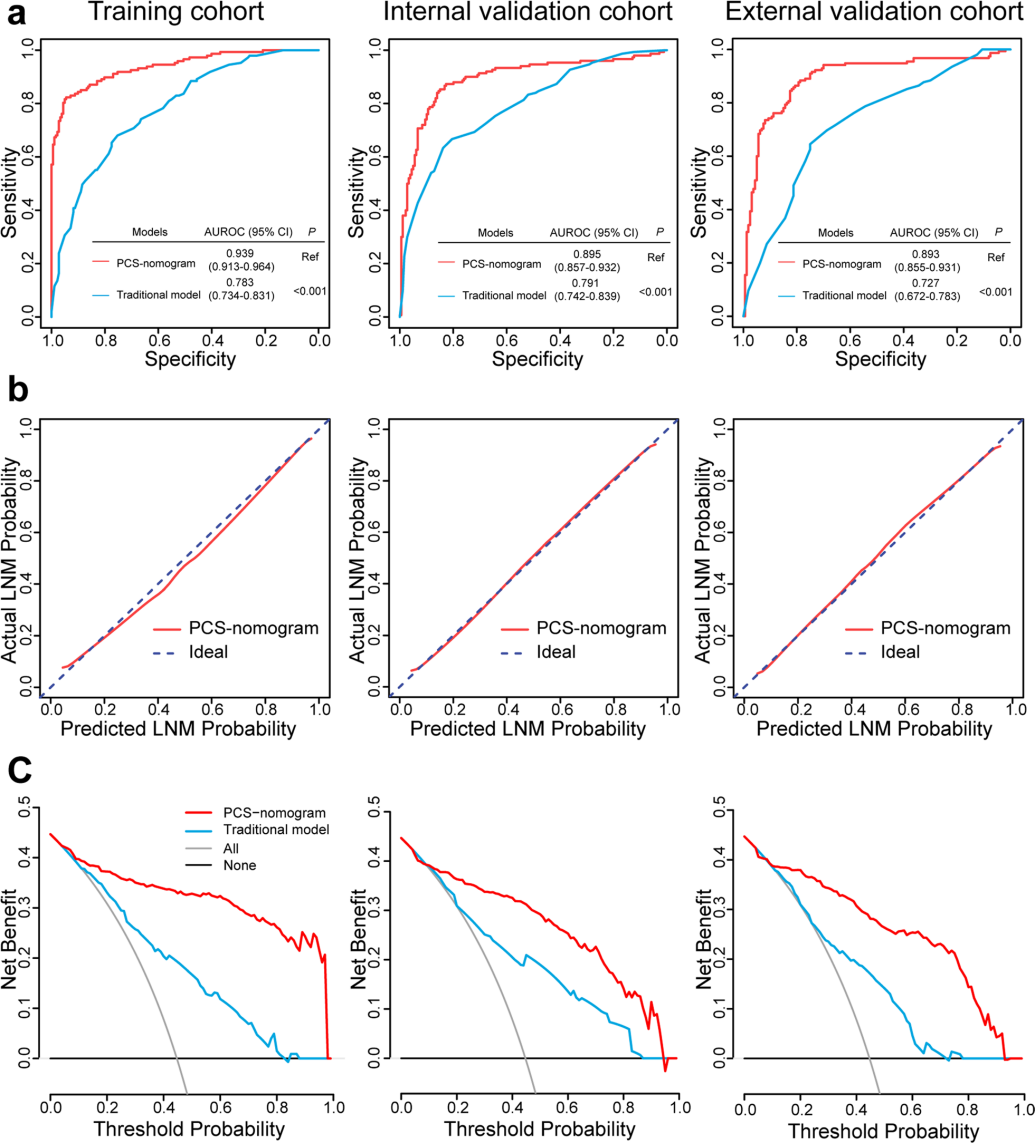
Performance of the PCS-nomogram to predict LNM. **a** The ROC curves of the PCS-nomogram and the traditional model to predict LNM in the training cohort and the internal and external validation cohorts. **b** The calibration curves of the PCS-nomogram in the training cohort and the internal and external validation cohorts. **c** DCA for the PCS-nomogram and the traditional model in each cohort. In the DCA curves, the y-axis measures the net benefit. The red line represents the PCS-nomogram, the blue line represents the traditional model, the gray line represents the assumption that all patients have LNM, and the black line represents the assumption that no patients have LNM. The results showed that if the threshold probability was > 0.10, using the PCS-nomogram to predict LNM could add more benefits than the traditional model. *PCS* pathomics-collagen signature, *LNM* lymph node metastasis, *AUROC* area under the receiver operating characteristic curve, *DCA* decision curve analysis

The PCS-nomogram showed satisfactory discrimination with an AUROC of 0.939 (95% CI, 0.913–0.964) in the training cohort. The calibration curves showed good agreement between the predicted and actual probability of LNM (Fig. 4). The Hosmer–Lemeshow test demonstrated *P* = 0.634, which suggested no departure from a good fit. Good discrimination and calibration were observed in the internal validation cohort [AUROC: 0.895 (95% CI, 0.857–0.932)] and in the external validation cohort [0.893 (95% CI, 0.855–0.931)] (Fig. 4).

### Clinical application value of the PCS-nomogram

The traditional model was developed based on the preoperative CEA level, lymphadenectasis on CT, VELIPI, and pT stage in the training cohort (Additional file 1: Table S4). The traditional model yielded AUROCs of 0.783 (95% CI, 0.734–0.831), 0.791 (95% CI, 0.742–0.839), and 0.727 (95% CI, 0.672–0.783) in the three cohorts. The PCS-nomogram showed a superior discrimination ability to the traditional model in the three cohorts (*P* < 0.001) (Fig. 4). DCA showed that if the threshold probability was > 0.10, using the PCS-nomogram to predict LNM could add more benefits than the traditional model (Fig. 4). In addition, the PCS-nomogram showed higher values of sensitivity, specificity, accuracy, PPV, and NPV in the three cohorts (Table 3). The corresponding NRI and IDI showed significantly increased classification accuracy of the PCS-nomogram compared with the traditional model for LNM prediction (all *P* < 0.05) (Additional file 1: Table S5).

**Table 3:** Predictive power of the PCS-nomogram and traditional clinicopathological model for LNM

Variables	AUROC	Sensitivity	Specificity	Accuracy	PPV	NPV
Training cohort						
PCS-nomogram	0.939 (0.913, 0.964)	82.3% (75.3%, 87.6%)	94.5% (90.2%, 97.0%)	89.1% (85.2%, 92.0%)	92.4% (86.5%, 95.8%)	86.9% (81.5%, 90.9%)
Traditional model	0.783 (0.734, 0.831)	68.0% (61.1%, 75.0%)	75.3% (68.5%, 81.0%)	72.0% (67.0%, 76.6%)	69.0% (61.0%, 75.9%)	74.5% (67.7%, 80.2%)
Internal validation cohort						
PCS-nomogram	0.895 (0.857, 0.932)	85.3% (78.8%, 90.1%)	84.9% (78.9%, 89.4%)	85.1% (80.9%, 88.5%)	82.6% (75.8%, 87.7%)	87.4% (81.6%, 91.5%)
Traditional model	0.791 (0.742, 0.839)	63.3% (55.4%, 70.6%)	83.8% (77.7%, 88.5%)	74.5% (69.5%, 78.9%)	76.6% (68.4%, 83.2%)	73.2% (66.7%, 78.8%)
External validation cohort						
PCS-nomogram	0.893 (0.855, 0.931)	88.4% (82.4%, 92.5%)	78.8% (71.8%, 84.4%)	83.5% (79.0%, 87.2%)	80.1% (73.5%, 85.4%)	87.5% (81.1%, 91.9%)
Traditional model	0.727 (0.672, 0.783)	64.5% (56.7%, 71.6%)	75.0% (67.8%, 81.1%)	69.8% (64.6%, 74.6%)	71.4% (63.5%, 78.3%)	68.6% (61.4%, 75.0%)

*LNM* lymph node metastasis, *AUROC* area under the receiver operating characteristic curve, *PCS* pathomics-collagen signature, *PPV* positive predictive value, *NPV* negative predictive value

**Fig. 5:**
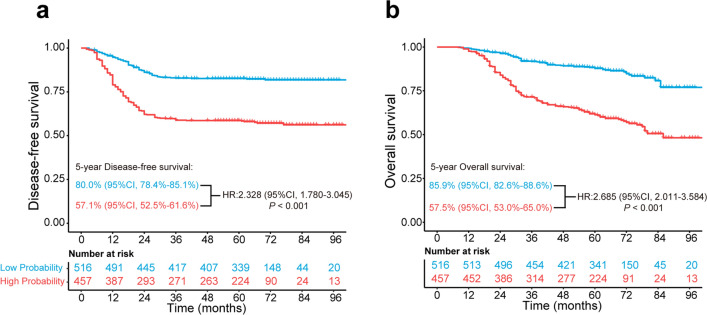
Kaplan–Meier analysis of disease-free survival and overall survival according to the nomogram-predicted subgroups of all patients. **a** Disease-free survival of all patients in the high- and low-probability LNM subgroups. **b** Overall survival of all patients in the high- and low-probability LNM subgroups. *LNM* lymph node metastasis, *HR* hazard ratio, *CI* confidence interval

The median follow-up was 61 months (IQR, 40–71) in all patients (n = 973), with a 5-year DFS of 70.3% (95% CI, 67.4%-73.1%) and a 5-year OS of 72.5% (95% CI, 69.6%-75.2%) (Additional file 1: Fig. S6). Kaplan–Meier analysis showed that patients in the PCS-nomogram-predicted low LNM probability subgroup had a favorable DFS compared with the high LNM probability subgroup [5-year DFS: low LNM probability, 80.0% (95% CI, 78.4%-85.1%); high LNM probability, 84.7% (52.5%-61.6%); log-rank *P* < 0.001] (Fig. 5a). Similar results for OS between patients in the low and high LNM probability subgroups were observed [5-year OS: low LNM probability, 85.9% (95% CI, 82.6%-88.6%); high LNM probability, 57.5% (53.0%-65.0%); log-rank *P* < 0.001] (Fig. 5b). Univariate and multivariable Cox regression showed that the nomogram-predicted LNM probability was an independent prognostic factor for DFS (HR, 2.328; 95% CI, 1.780–3.045) and OS (HR, 2.685; 95% CI, 2.011–3.584) after adjusting for clinicopathological risk factors in CRC patients (Additional file 1: Table S6).

### PCS***-nomograms for predicting LNM at station Nos. 1, 2, and 3***

Univariate and multivariable logistic regression analyses indicated that the PCS was still an independent predictor for LNM at station Nos. 1, 2, and 3 in the training cohort (Additional file 1: Tables S7–S9). Then, we developed three PCS-nomograms to predict LNM at the three nodal stations (Additional file 1: Figs S7–S9). The PCS-nomograms indicated satisfactory performance of prediction outcomes, with AUROCs of 0.939 (95% CI, 0.913–0.965) for LNM at station No. 1, 0.885 (95% CI, 0.842–0.904) for LNM at station No. 2, and 0.849 (95% CI, 0.778–0.919) for LNM at station No. 3 in the training cohort. The PCS-nomograms also showed satisfactory prediction performance outcomes, with AUROCs of 0.837–0.902 in the internal validation cohort and 0.851–0.895 in the external validation cohort for LNM at the three nodal stations (Additional file 1: Tables S10–S12). Correspondingly, we also constructed three traditional models for comparison with the PCS-nomograms (Additional file 1: Tables S13–S15). The results show that the PCS-nomograms were significantly superior to the traditional models for LNM prediction at the three nodal stations (Additional file 1: Tables S10–S12, S16–S18, and Figs. S10–S12).

## Discussion

In this study, we constructed a PCS that integrated 11 pathomics features from digital H&E images and 9 collagen features from multiphoton images to illuminate the relationship between the tumor with its microenvironment and LNM. We found that PCS was significantly associated with LNM. Then, we developed and validated a PCS-nomogram for predicting individual LNM in CRC patients. The PCS-nomogram demonstrated satisfactory discrimination and calibration in the three cohorts. In addition, compared with the traditional model, the PCS-nomogram displayed better predictive performance for LNM.

Traditional H&E stained slides are the gold standard for disease diagnosis. In the trend of digital medicine, whole glass slide imaging has been gradually used in clinical practice and stored in the form of a digital H&E image [41, 42]. Importantly, digital H&E imaging is not only a powerful tool for tumor diagnosis but also contains a wealth of pathological information. Some studies have proven that quantitative pathological information can be applied for disease diagnosis, risk stratification, and outcome prediction via an appropriate feature extraction method, i.e., pathomics [14, 17]. Cao R and his colleagues reported that pathomics could be used to predict microsatellite instability in CRC [43]. Additionally, pathomics could serve as a prognostic marker for evaluating the prognosis of patients with clear cell renal cell carcinoma [17]. Moreover, pathomics could seamlessly integrate into other omics methods to improve model performance, including the assessment of lung metastasis prognosis in CRC patients and the evaluation of treatment response in rectal cancer patients after neoadjuvant chemoradiotherapy [16, 44]. These investigations revealed that digital pathomics features can reflect underlying molecular characteristics or genetic patterns, which could complement tumor heterogeneity and increase the predictive ability of existing models [36, 45, 46]. CellProfiler is an easy-to-use and reproducible tool to automatically measure various phenotypes from biological images with satisfactory performance [31, 47–49]. Therefore, CellProfiler was used to extract pathomics features from digital H&E images in our study.

The extracellular matrix (ECM) constitutes the scaffold of the TME, which regulates tumor behavior [18, 19]. Collagen is the main component and performs the main function of the ECM. Emerging evidence has shown that the collagen structure in the TME is significantly associated with tumor biological behavior, including metastasis [50, 51]. However, traditional H&E images cannot be used to illuminate collagen structure alterations in the TME. MPI can visualize collagen structure at the subcellular level [20]. Importantly, our previous studies constructed a stable framework that can achieve precise quantification features from multiphoton images to evaluate the relationship between collagen features and various outcomes [27, 28, 52]. Thus, we believe that integrating pathomics and collagen features can provide a comprehensive interpretation of the relationship between the tumor with its microenvironment and LNM.

After obtaining high-dimensional pathomics features and collagen features, it is important to use reasonable machine learning algorithms to build predictive models. LASSO is an effective algorithm to deal with high-dimensional data and obtain a linear combination of selected features to calculate the PSC [33, 34]. The penalty parameter λ of LASSO controls the strength of the penalty. When λ is reduced and the penalty is relaxed, the model incorporates more parameters, thereby increasing its complexity and the risk of overfitting. Conversely, when λ is increased and the penalty is strong, the model includes fewer parameters, potentially impacting its accuracy. Therefore, the optimal value of λ was determined by tenfold cross-validation with 1—standard error criterion, which is the balance between the accuracy and complexity of the model. As a result, a total of 256 candidate features, including 114 pathomics features and 142 collagen features, were reduced to the 20 most predictive features to construct the PCS. The PCS that combines 11 pathomics features and 9 collagen features showed satisfactory discrimination in the training cohort (AUROC = 0.939), which was then validated in the internal (AUROC = 0.895) and external (AUROC = 0.893) validation cohorts. In addition, the PCS showed superior prediction performance over the pathomics signature and collagen signature in the three cohorts. Although the Lasso regression is applicable in many situations, it also has several limitations. Lasso regression may encounter challenges when the number of parameters significantly surpasses the number of patients; moreover, if there are two or more highly collinear parameters, Lasso regression will randomly select one, which is not conducive to data interpretation [53, 54].

LNM is critical for therapeutic decision-making and predicting the prognosis of patients with CRC. Currently, the overall accuracy of medical imaging for lymph node status remains unsatisfactory [6, 10]. Lymphadenectasis on CT ≥ 10 mm was an independent predictor for LNM. The traditional model based on lymphadenectasis on CT and three other risk factors for comparison with the PCS-nomogram. The PCS-nomogram was more powerful performance than the traditional model in evaluating the risk of LNM in CRC in three cohorts. Moreover, the PCS was still an independent predictor of LNM at station Nos. 1, 2, and 3. Then, we built three PCS-nomograms to predict LNM at the three nodal stations. The PCS-nomogram displayed AUROCs of 0.849–0.939 for the training cohort, 0.837–0.902 for the internal validation cohort, and 0.851–0.895 for the external validation cohorts in the three nodal stations. Similarly, the PCS-nomograms performed better than the traditional model. Thus, PCS-nomograms have potential clinical applications to assist clinical decisions. This work provided a new method for assessing lymph node status and suggests the potential for utilizing biopsy tissues for predicting lymph node status preoperatively to assist in clinical decision-making. To effectively incorporate PCS in guiding decisions regarding the optimal course of surgery or neoadjuvant treatment in a clinical setting, it is imperative to facilitate its transfer to biopsy tissues. In patients with early-stage CRC with a low probability of LNM, surgical approaches include endoscopic resection and local excision. Conversely, for patients with a high probability of LNM, a more suitable option may involve radical resection combined with lymph node dissection. Notably, in rectal cancer, the presence of LNM indicates an advanced stage, where neoadjuvant treatment is the recommended therapeutic approach.

Despite the exploratory findings of our study, there are still some limitations. First, this was a retrospective multicenter study, and selection bias could not be avoided. To address this, we will carry out a prospective, large-sample, multicenter study to further validate the robustness of the PCS-nomograms. Second, manually delineating the representative area of tumor invasion is a time-consuming and labor-intensive task. Consequently, our plan entails establishing a fully automated system in the future. Third, we confirmed the correlation between LNM and PCS from the specimens. Our next step involved transferring the model to preoperative biopsy tissues. Finally, genetic data are important for comprehensive analysis, and further work should explore the biological underpinnings of PCS through genomic analysis.

## Conclusions

The PCS based on pathomics features and collagen features is significantly associated with LNM, and the PCS-nomogram has the potential to be a useful tool for predicting individual LNM in CRC patients.

### Supplementary Information

**Additional file 1: Figure S1.** Recruitment pathway for the patient in this study. **Figure S2.** Feature selection and pathomics-collagen signature construction. **Figure S3.** Feature selection and single-modality signature construction. **Figure S4.** ROC curves of the pathomics-collagen signature and other predictors. **Figure S5.** Multicollinearity of the predictors of the PCS-nomogram. **Figure S6.** Kaplan−Meier survival analysis in all patients. **Figure S7.** PCS-nomogram to predict LNM at station No. 1 in patients with CRC. **Figure S8.** PCS-nomogram to predict LNM at station No. 2 in patients with CRC. **Figure S9.** PCS-nomogram to predict LNM at station No. 3 in patients with CRC. **Figure S10.** Performance of the PCS-nomogram to predict LNM at station No. 1. **Figure S11.** Performance of the PCS-nomogram to predict LNM at station No. 2. **Figure S12.** Performance of the PCS-nomogram to predict LNM at station No. 3. **Table S1.** Extracted 114 pathomics features. **Table S2.** Extracted 142 collagen features. **Table S3.** Stratified analysis of the association between the PCS and LNM in the training, internal validation, and external validation cohorts. **Table S4.** Univariate and multivariable analyses of the predictors of LNM without the pathomics-collagen signature in the training cohort. **Table S5.** NRI and IDI test for the prediction of LNM improvements of the PCS-nomogram compared with the traditional model. **Table S6.** Cox regression analysis of the predictors of survival in all patients. **Table S7.** Univariate and multivariable analyses of the predictors of LNM at station No. 1 in the training cohort. **Table S8.** Univariate and multivariable analyses of the predictors of LNM at station No. 2 in the training cohort. **Table S9.** Univariate and multivariable analyses of the predictors of LNM at station No. 3 in the training cohort. **Table S10.** Predictive power of LNM at station No. 1 between the PCS-nomogram and traditional model. **Table S11.** Predictive power of LNM at station No. 2 between the PCS-nomogram and traditional model. **Table S12.** Predictive power of LNM at station No. 3 between the PCS-nomogram and traditional model. **Table S13.** Univariate and multivariable analyses of the predictors of LNM at station No. 1 without pathomics-collagen signature in the training cohort. **Table S14.** Univariate and multivariable analyses of the predictors of LNM at station No. 2 without pathomics-collagen signature in the training cohort. **Table S15.** Univariate and multivariable analyses of the predictors of LNM at station No. 3 without pathomics-collagen signature in the training cohort. **Table S16.** NRI and IDI test for prediction of LNM at station No 1. improvements of PCS-nomogram compared with the traditional model. **Table S17.** NRI and IDI test for prediction of LNM at station No. 2 improvements of PCS-nomogram compared with the traditional model. **Table S18.** NRI and IDI test for prediction of LNM at station No. 3 improvements of PCS-nomogram compared with the traditional model.

## Data Availability

The data that support the findings of this study are available from the corresponding author upon reasonable request.

## Abbreviations

AUROC: Area under the receiver operating characteristic curve
CI: Confidence interval
CRC: Colorectal cancer
DCA: Decision curve analysis
DFS: Disease-free survival
H&E: Hematoxylin and eosin
HR: Hazard ratio
IDI: Integrated discrimination improvement
IQR: Interquartile
LASSO: Least absolute shrinkage and selection operator
LNM: Lymph node metastasis
NPV: Negative predictive value
NRI: Net reclassification improvement
OS: Overall survival
OR: Odds ratio
PCS: Pathomics-collagen signature
PPV: Positive predictive value
ROI: Regions of interest
STARD: Standards for Reporting Diagnostic Accuracy
TME: Tumor microenvironment
SHG: Second harmonic generation
TPEF: 2-Photon excitation fluorescence
VELIPI: Venous emboli and/or lymphatic invasion, and/or perineural invasion
VIF: Variance inflation factor
