# Supplementary material for: Association of the pathomics-collagen signature with lymph node metastasis in colorectal cancer: a retrospective multicenter study
Source: J Transl Med. 2024 Jan 25;22:103. doi: 10.1186/s12967-024-04851-2 (PMC10811897; doi:10.1186/s12967-024-04851-2)
Supplement: Supplementary file 1 — Additional file 1: Figure S1. Recruitment pathway for the patient in this study. Figure S2. Feature selection and pathomics-collagen signature construction. Figure S3. Feature selection and single-modality signature construction. Figure S4. ROC curves of the pathomics-collagen signature and other predictors. Figure S5. Multicollinearity of the predictors of the PCS-nomogram. Figure S6. Kaplan−Meier survival analysis in all patients. Figure S7. PCS-nomogram to predict LNM at station No. 1 in patients with CRC. Figure S8. PCS-nomogram to predict LNM at station No. 2 in patients with CRC. Figure S9. PCS-nomogram to predict LNM at station No. 3 in patients with CRC. Figure S10. Performance of the PCS-nomogram to predict LNM at station No. 1. Figure S11. Performance of the PCS-nomogram to predict LNM at station No. 2. Figure S12. Performance of the PCS-nomogram to predict LNM at station No. 3. Table S1. Extracted 114 pathomics features. Table S2. Extracted 142 collagen features. Table S3. Stratified analysis of the association between the PCS and LNM in the training, internal validation, and external validation cohorts. Table S4. Univariate and multivariable analyses of the predictors of LNM without the pathomics-collagen signature in the training cohort. Table S5. NRI and IDI test for the prediction of LNM improvements of the PCS-nomogram compared with the traditional model. Table S6. Cox regression analysis of the predictors of survival in all patients. Table S7. Univariate and multivariable analyses of the predictors of LNM at station No. 1 in the training cohort. Table S8. Univariate and multivariable analyses of the predictors of LNM at station No. 2 in the training cohort. Table S9. Univariate and multivariable analyses of the predictors of LNM at station No. 3 in the training cohort. Table S10. Predictive power of LNM at station No. 1 between the PCS-nomogram and traditional model. Table S11. Predictive power of LNM at station No. 2 between the PCS-nomogram and tr [file 12967_2024_4851_MOESM1_ESM.docx]

# Additional file 1

[Supplementary Materials 1](#_Toc129446964)

[I. Supplementary Methods 3](#_Toc129446965)

[1. Pipeline for the extraction of pathomics features using CellProfiler. 3](#_Toc129446966)

[2. Multiphoton imaging system. 6](#_Toc129446967)

[3. Collagen feature extraction. 7](#_Toc129446968)

[4. Construction of the pathomics-collagen signature using LASSO regression. 8](#_Toc129446969)

[5. Decision curve analysis of the PCS-nomogram. 9](#_Toc129446970)

[6. Integrated discrimination improvement and net reclassification improvement. 10](#_Toc129446971)

[II. Supplementary Results 11](#_Toc129446972)

[III. Supplementary Figures 12](#_Toc129446973)

[Figure S1. Recruitment pathway for the patient in this study. 12](#_Toc129446974)

[Figure S2. Feature selection and pathomics-collagen signature construction. 13](#_Toc129446975)

[Figure S3. Feature selection and single-modality signature construction. 14](#_Toc129446976)

[Figure S4. ROC curves of the pathomics-collagen signature and other predictors. 15](#_Toc129446977)

[Figure S5. Multicollinearity of the predictors of the PCS-nomogram. 16](#_Toc129446978)

[Figure S6. Kaplan−Meier survival analysis in all patients. 17](#_Toc129446979)

[Figure S7. PCS-nomogram to predict LNM at station No. 1 in patients with CRC. 18](#_Toc129446980)

[Figure S8. PCS-nomogram to predict LNM at station No. 2 in patients with CRC. 19](#_Toc129446981)

[Figure S9. PCS-nomogram to predict LNM at station No. 3 in patients with CRC. 20](#_Toc129446982)

[Figure S10. Performance of the PCS-nomogram to predict LNM at station No. 1. 21](#_Toc129446983)

[Figure S11. Performance of the PCS-nomogram to predict LNM at station No. 2. 22](#_Toc129446984)

[Figure S12. Performance of the PCS-nomogram to predict LNM at station No. 3. 23](#_Toc129446985)

[IV. Supplementary Tables 24](#_Toc129446986)

[Table S1. Extracted 114 pathomics features. 24](#_Toc129446987)

[Table S2. Extracted 142 collagen features. 25](#_Toc129446988)

[Table S3. Stratified analysis of the association between the PCS and LNM in the training, internal validation, and external validation cohorts. 26](#_Toc129446989)

[Table S4. Univariate and multivariable analyses of the predictors of LNM without the pathomics-collagen signature in the training cohort. 27](#_Toc129446990)

[Table S5. NRI and IDI test for the prediction of LNM improvements of the PCS-nomogram compared with the traditional model. 28](#_Toc129446991)

[Table S6. Cox regression analysis of the predictors of survival in all patients. 29](#_Toc129446992)

[Table S7. Univariate and multivariable analyses of the predictors of LNM at station No. 1 in the training cohort. 30](#_Toc129446993)

[Table S8. Univariate and multivariable analyses of the predictors of LNM at station No. 2 in the training cohort. 31](#_Toc129446994)

[Table S9. Univariate and multivariable analyses of the predictors of LNM at station No. 3 in the training cohort. 32](#_Toc129446995)

[Table S10. Predictive power of LNM at station No. 1 between the PCS-nomogram and traditional model. 33](#_Toc129446996)

[Table S11. Predictive power of LNM at station No. 2 between the PCS-nomogram and traditional model. 34](#_Toc129446997)

[Table S12. Predictive power of LNM at station No. 3 between the PCS-nomogram and traditional model. 35](#_Toc129446998)

[Table S13. Univariate and multivariable analyses of the predictors of LNM at station No. 1 without pathomics-collagen signature in the training cohort. 36](#_Toc129446999)

[Table S14. Univariate and multivariable analyses of the predictors of LNM at station No. 2 without pathomics-collagen signature in the training cohort. 37](#_Toc129447000)

[Table S15. Univariate and multivariable analyses of the predictors of LNM at station No. 3 without pathomics-collagen signature in the training cohort. 38](#_Toc129447001)

[Table S16. NRI and IDI test for prediction of LNM at station No 1. improvements of PCS-nomogram compared with the traditional model. 39](#_Toc129447002)

[Table S17. NRI and IDI test for prediction of LNM at station No. 2 improvements of PCS-nomogram compared with the traditional model. 39](#_Toc129447003)

[Table S18. NRI and IDI test for prediction of LNM at station No. 3 improvements of PCS-nomogram compared with the traditional model. 39](#_Toc129447004)

[V. Supplementary References 40](#_Toc129447005)

# I. Supplementary Methods

## 1. Pipeline for the extraction of pathomics features using CellProfiler.

A total of 114 pathomics features were extracted, including 57 image quality features, 9 image colocalization features, and 48 image granularity features (**Table S1**).

CellProfiler is free, open-source software for quantitative analysis of biological images (<https://cellprofiler.org/>) [1-4]. The hematoxylin and eosin (H&E)-stained images were split into hematoxylin-stained and eosin-stained grayscale images using the *“UnmixColors”* module [5]. The digital H&E-stained images were also converted to grayscale images using the *“ColorToGray”* module based on the *“Combine”* method for further analysis.

**(1) Image quality features**

First, the features that indicated the image quality of the greyscale H&E, haematoxylin and eosin images were assessed by using the *“MeasureImageQuality”* module.

*IntegratedIntensity:* The sum of the pixel intensities within an object.

*MeanIntensity:* The average pixel intensity within an object.

*StdIntensity:* The standard deviation of the pixel intensities within an object.

*MaxIntensity:* The maximal pixel intensity within an object.

*MinIntensity:* The minimal pixel intensity within an object.

*IntegratedIntensityEdge:* The sum of the edge pixel intensities of an object.

*MeanIntensityEdge:* The average edge pixel intensity of an object.

*StdIntensityEdge:* The standard deviation of the edge pixel intensities of an object.

*MaxIntensityEdge:* The maximal edge pixel intensity of an object.

*MinIntensityEdge:* The minimal edge pixel intensity of an object.

*MassDisplacement:* The distance between the centers of gravity in the gray-level representation of the object and the binary representation of the object.

*LowerQuartileIntensity:* The intensity value of the pixel for which 25% of the pixels in the object have lower values.

*MedianIntensity:* The median intensity value within the object.

*MADIntensity:* The median absolute deviation (MAD) value of the intensities within the object. The MAD is defined as the median(|xi - median(x)|).

*UpperQuartileIntensity:* The intensity value of the pixel for which 75% of the pixels in the object have lower values.

*Location_CenterMassIntensity_X, Location_CenterMassIntensity_Y:* The (X,Y) coordinates of the intensity-weighted centroid (= center of mass = first moment) of all pixels within the object.

*Location_MaxIntensity_X, Location_MaxIntensity_Y:* The (X,Y) coordinates of the pixel with the maximum intensity within the object.

**(2) Image colocalization features**

Subsequently, the colocalization and correlation between intensities in each hematoxylin image and eosin image were calculated on a pixel-by-pixel basis across an entire image by using the *“MeasureColocalization”* module [6].

*Correlation:* The correlation between a pair of hematoxylin (H)- and eosin (E)-stained images was calculated as Pearson’s correlation coefficient. The calculation formula is covariance (H, E)/[std(H) × std(E)].

*Slope:* The slope of the least-squares regression between a pair of H and E images. Calculated using the model *α* × H + *β* = E, where *α* is the slope.

*Overlap coefficient:* The overlap coefficient is a modification of Pearson’s correlation coefficient in which the average intensity values of the pixels are not subtracted from the original intensity values. For a pair of H and E images, the overlap coefficient calculation formula is sum (H_i_ × E_i_)/sqrt (sum (H_i_× H_i_) × sum (E_i_ × E_i_)).

*Manders coefficient:* The Manders coefficient for a pair of H and E images is calculated as M1 = sum (H_i__coloc)/sum (H_i_) and M2 = sum (E_i__coloc)/sum (E_i_), where H_i__coloc = H_i_ when E_i_ > 0 or 0 otherwise, and E_i__coloc = E_i_ when H_i_ > 0 or 0 otherwise.

*Manders coefficient (Costes automated threshold):* Costes’ automated threshold estimates the maximum threshold of intensity for each image based on the *correlation* value. The Manders coefficient is applied on thresholded images as H_i__coloc = H_i_ when E_i_ > E_thr_ and E_i__coloc = E_i_ when H_i_ > H_thr_, where E_thr_ and H_thr_ are thresholds calculated using Costes’ automated threshold method.

*Rank weighted colocalization coefficient:* The rank weighted colocalization (RWC) coefficient for a pair of H and E images is measured as RWC1 = sum(H_i__coloc*W_i_)/sum(H_i_) and RWC2 = sum(E_i__coloc*W_i_)/sum(E_i_); in this formula, W_i_ is the weight defined as W_i_ = (H_max_ - D_i_)_/Rmax_, where R_max_ is the maximum ranks among H and E based on the max intensity, and D_i_ = abs(rank(H_i_) - rank(E_i_)) (absolute difference in ranks between H and R) and H_i__coloc = H_i_ when E_i_ > 0 or 0 otherwise and E_i__coloc = E_i_ when H_i_ >0 or 0 otherwise.

**(3) Image granularity features**

In addition, the granularity features of each image were calculated using the *“MeasureGranularity”* module, which outputted spectra of size measurements of the textures in the image, with a granular spectrum range of 16 [7-10].

*Granularity:* The module returns one measurement for each instance of the granularity spectrum set in the range of the granular spectrum.

## 2. Multiphoton imaging system.

The multiphoton microscopic imaging system used in this work has been described previously [11]. In brief, an upright microscope (LSM 880, Zeiss, Germany) equipped with a mode-locked femtosecond Ti: sapphire laser (Chameleon Ultra, Coherent) was used to obtain high-resolution images. In this study, 810 nm linearly polarized light was selected for nonlinear optical imaging, and a Plan-Apochromat ×20 objective (NA = 0.8, Zeiss, Germany) was used for focusing the excitation beam into the samples. A 32-channel GaAsP photomultiplier tube array detector was used for collecting second harmonic generation (SHG) signals (green color) in the wavelength range of 387 to 409 nm, and for the collection of two-photon excitation fluorescence (TPEF) signals (red color) in the wavelength range from 430 to 708 nm. To obtain a large-scale image, a fine focusing stage is used to translate the samples, and each large-scale image was stitched together from a series of x-y scan images. Each x-y scan image contains 512 × 512 pixels with a data depth of 12 bits. Then, multiphoton images were compared to the H&E images for histological evaluation.

## 3. Collagen feature extraction.

A total of 142 collagen features were extracted, including 8 morphological features and 134 texture features (**Table S2**).

***Morphological features***

Eight morphological features were extracted, namely, the collagen area, fiber number, length, width, straightness, crosslink density, crosslink space, and orientation. The SHG image was first segmented into collagen pixels and background pixels using the Gaussian mixture model method [12]. The binary collagen mask image was then processed using a fiber network extraction algorithm [13] to trace each collagen fiber in the image and to identify cross-link points, which are defined as connecting points between two or more fibers. Moreover, we quantified an orientation index to reflect the collagen alignment based on Fourier transform spectra [14].

***Intensity features***

For intensity features, a histogram-based approach was used. The mean, variation, skewness, kurtosis, energy, and entropy were calculated from the histogram of the SHG pixel intensity distribution.

***Texture features***

We also included 80 gray-level co-occurrence matrix (GLCM)-based texture features and 48 Gabor wavelet transform features in the analysis [15, 16]. The contrast, correlation, energy, and homogeneity were calculated from the GLCM with five different displacements of pixels at 1, 2, 3, 4, and 5 and four different directions at 0, 45, 90, and 135 degrees. To calculate the Gabor wavelet transform features, we convolved the SHG image with Gabor filters at five different scales and six different orientations, and the mean and variance of the magnitude of the convolution over the image at each setting were calculated.

4. Construction of the pathomics-collagen signature using LASSO regression.

The least absolute shrinkage and selection operator (LASSO) is a commonly used high-dimensional predictive regression method whose basic idea is to use the L1 penalty to accurately shrink certain regression coefficients to zero and further obtain an interpretable model [17, 18]. Its function expression is as follows:

$$\sum_{i=1}^{N} \left[ y_{i}-g\left( \sum_{j=1}^{m} \beta_{j}x_{ij}+\beta_{0} \right) \right]^{2}+\lambda\sum_{j=1}^{m} \left| \beta_{j} \right|$$

where *y_i_* (*y* = 0 or 1) is the outcome (LNM or non-LNM) of patient *i*, *N* is the number of patients, *g* is the sigmoid function, *m* is the number of selection features used in the model, *β_j_* is the model parameter, *x_ij_* is the *j*_th_ (*j* = 1, 2, …, *d*) pathomics and collagen feature of the *i_th_* patient, and λ is the penalty parameter. The sigmoid function *g* is defined as follows:

$$g\left( z \right)= \frac{1}{1+e^{-z}}$$

The penalty parameter λ (also called the adjustment parameter) controls the amount of contraction. The larger the λ value is, the fewer the number of greater predictor contributions to the model are ​​selected. LASSO has been extended and widely used in logistic regression models for high-dimensional data analysis. In addition, LASSO can also be used to optimize the selection of markers in high-dimensional data to avoid overfitting by controlling the complexity of the model. Hence, we adopted the penalized logistic regression model with the LASSO penalty to simultaneously achieve shrinkage and variable selection. Ten-time cross-validations were used to determine the optimal values of λ via 1-SE (standard error) criteria. As a result, a value λ = 0.02469578 with log (λ) = -3.701123 was selected. The optimal tuning parameter resulted in 20 nonzero coefficients, which were used to construct the pathomics-collagen signature (**Figure S2**).

5. Decision curve analysis of the PCS-nomogram.

Decision curve analysis (DCA) is a simple and easy-to-understand mathematical model that judges the availability and benefits of the prediction model by calculating the net benefits under different threshold probabilities [19-21].

In this study, DCA was used to evaluate the prediction model for lymph node metastasis. By analyzing the actual results and model predictions, the net benefit was calculated using the following formula:

$$\text{Net benefit=}\frac{\text{True positive count}}{\text{n}}\text{-}\frac{\text{F}\text{al}\text{se positive count}}{\text{n}}\left( \frac{\text{P}_{\text{t}}}{\text{1-}\text{P}_{\text{t}}} \right)$$

In this formula, *P_t_* is the threshold probability of lymph node metastasis, and *n* is the total number of patients.

## 6. Integrated discrimination improvement and net reclassification improvement.

Integrated discrimination improvement (IDI) and net reclassification improvement (NRI) are used to compare the diagnostic capabilities of two prediction models and whether one model improves the diagnostic accuracy of the other. Therefore, IDI and NRI are receiving more attention from experts [22, 23]. The calculation formulas of IDI and NRI are as follows:

IDI =（IS_new_ – IS_old_）-（IP_new_ – IP_old_）

NRI =（IS_new_- IS_old_）+（IP_new_ - IP_old_）

IS: Integral sensitivity over all possible cutoff values

IP: 1-specificity under different classification threshold settings

The larger the IDI or NRI is, the better the prediction ability of the new model. If IDI or NRI is > 0, it is a positive improvement, indicating that the prediction ability of the new model is improved compared with the old model. If IDI or NRI is < 0, it is considered that the prediction ability of the new model is decreased compared with the old model. If IDI or NRI is = 0, it is considered that the new model is not improved.

# II. Supplementary Results

Pathomics-collagen signature = 0.142445 × IntegratedIntensity_Haematoxylin

- 9.987692 × LowerQuartileIntensity_H&E

+ 16.277736 × Manders Coefficient_Eosin

- 0.457274 × Granularity_ Hematoxylin_6

+ 0.191150 × Granularity_ Hematoxylin_16

+ 1.727374 × Granularity_ Eosin_1

+ 0.723935 × Granularity_ Eosin_4

+ 0.102639 × Granularity_ Eosin_5

- 0.034180 × Granularity_ H&E _6

- 0.014034 × Granularity_ H&E _11

+ 0.193117 × Granularity_ H&E _14

+ 6.943020 × Collagen Fiber Width

+ 1.542639 × Collagen Cross-link Density

- 2.070072 × Hisogram Skewness

- 2.914300 × GLCM_ Angle 0°_Displacement 1_Contrast

+ 0.000102 × GLCM_ Angle 0°_Displacement 1_Homogeneity

+ 3.315868 × GLCM_Angle 0°_Displacement 3_Contrast

+ 2.491848 × Gabor_Scale 2_Oritation 4_Variance

+ 0.560772 × Gabor_Scale 3_Oritation 2_Variance

+ 1.542134 × Gabor_Scale 4_Oritation 5_Variance

- 70.052120

# III. Supplementary Figures


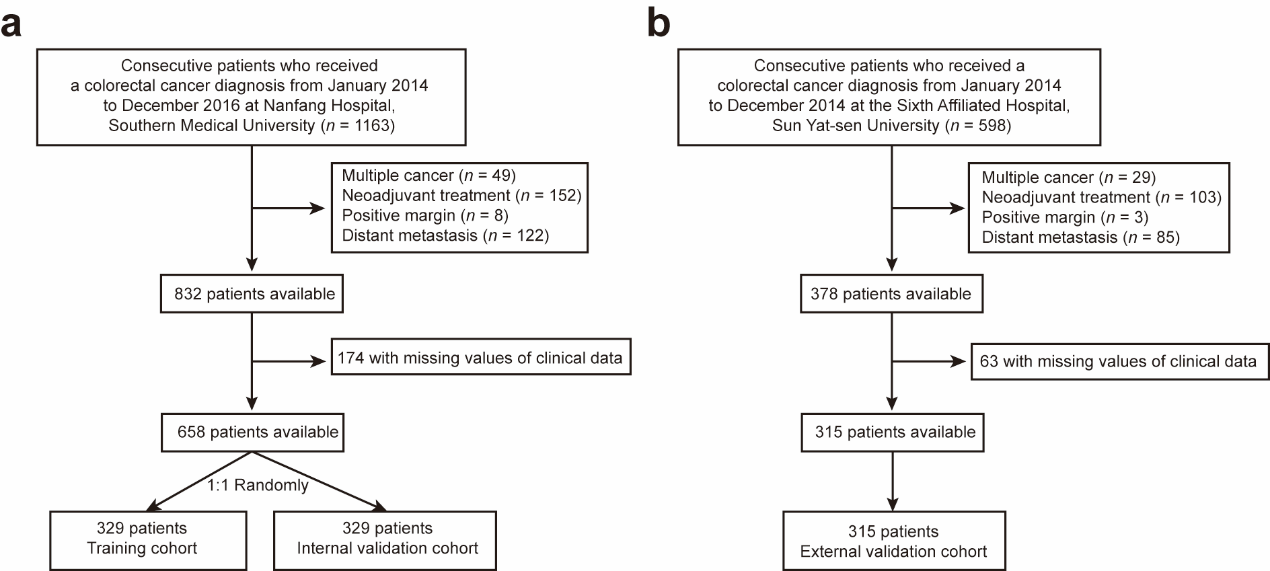


## Figure S1. Recruitment pathway for the patient in this study.


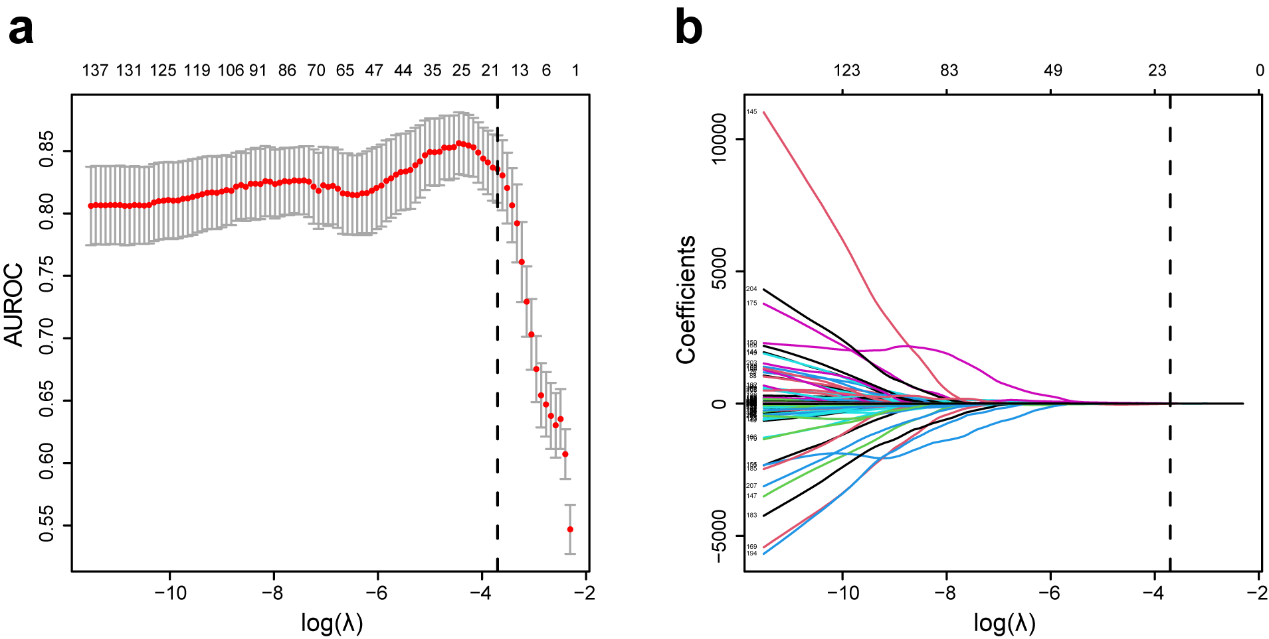


## Figure S2. Feature selection and pathomics-collagen signature construction.

**(a)** Selection of the optimal penalty parameter (λ) that is conducted by 10-fold cross-validation based on 1 standard error of the minimum criteria (right). The AUROC is plotted versus log (λ). The optimal λ value of 0.02469578 with log (λ) = -3.701123 is selected. **(b)** Distribution of the coefficients of 256 features in the LASSO logistic regression. The dotted vertical line is the optimal λ value of -3.701123 with 20 nonzero coefficients to construct the pathomics-collagen signature. *Abbreviations:* AUROC, area under the receiver operating characteristic curve; LASSO, least absolute shrinkage and selection operator.


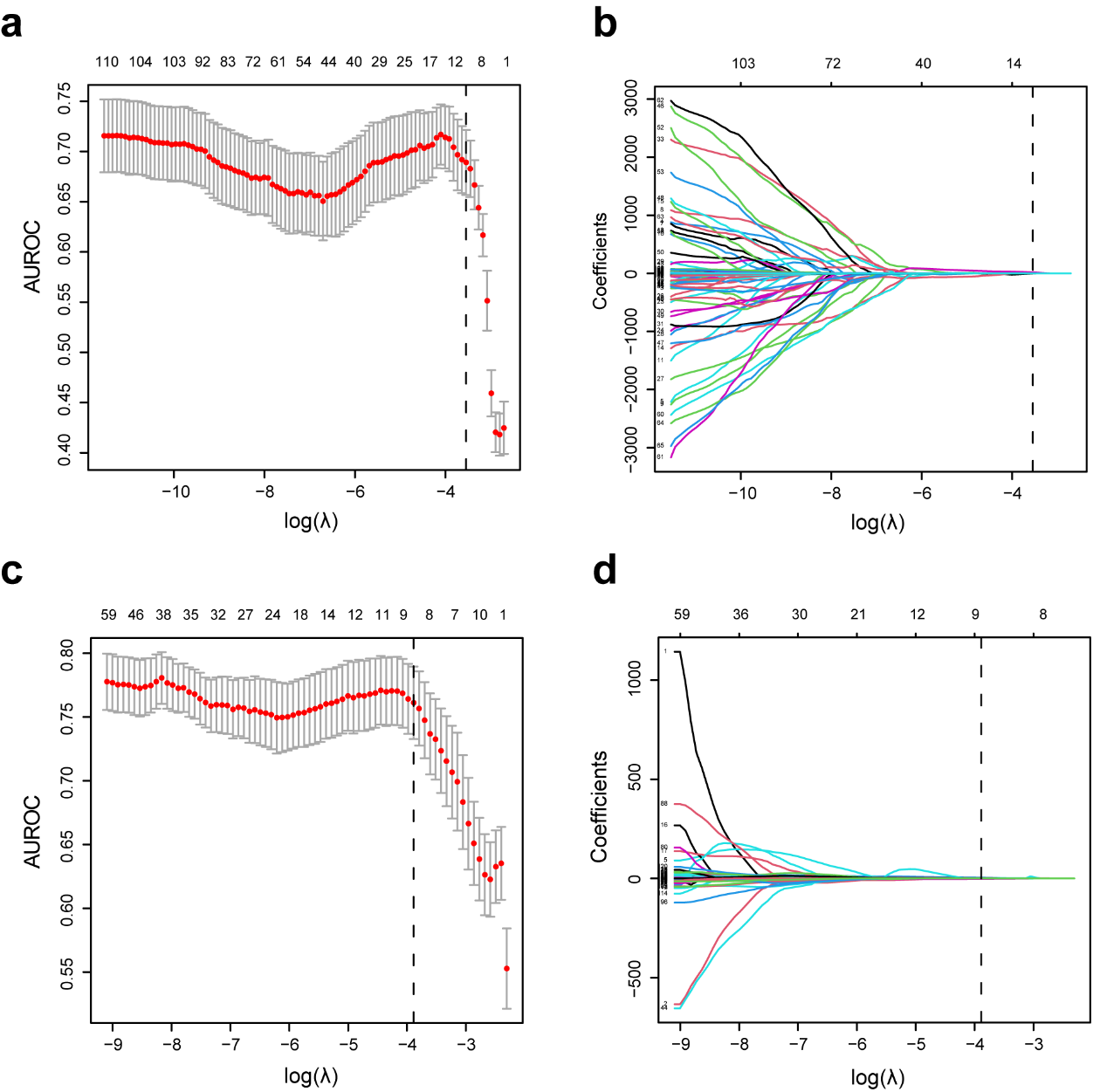


## Figure S3. Feature selection and single-modality signature construction.

**(a)** Selection of the optimal penalty parameter (λ) and (b) distribution of the coefficients of 114 pathomics features in LASSO logistic regression to construct the pathomics signature. **(c)** Selection of the optimal penalty parameter (λ) and (d) distribution of the coefficients of 142 collagen features in the LASSO logistic regression to construct the collagen signature. *Abbreviations:* AUROC, area under the receiver operating characteristic curve; LASSO, least absolute shrinkage and selection operator.


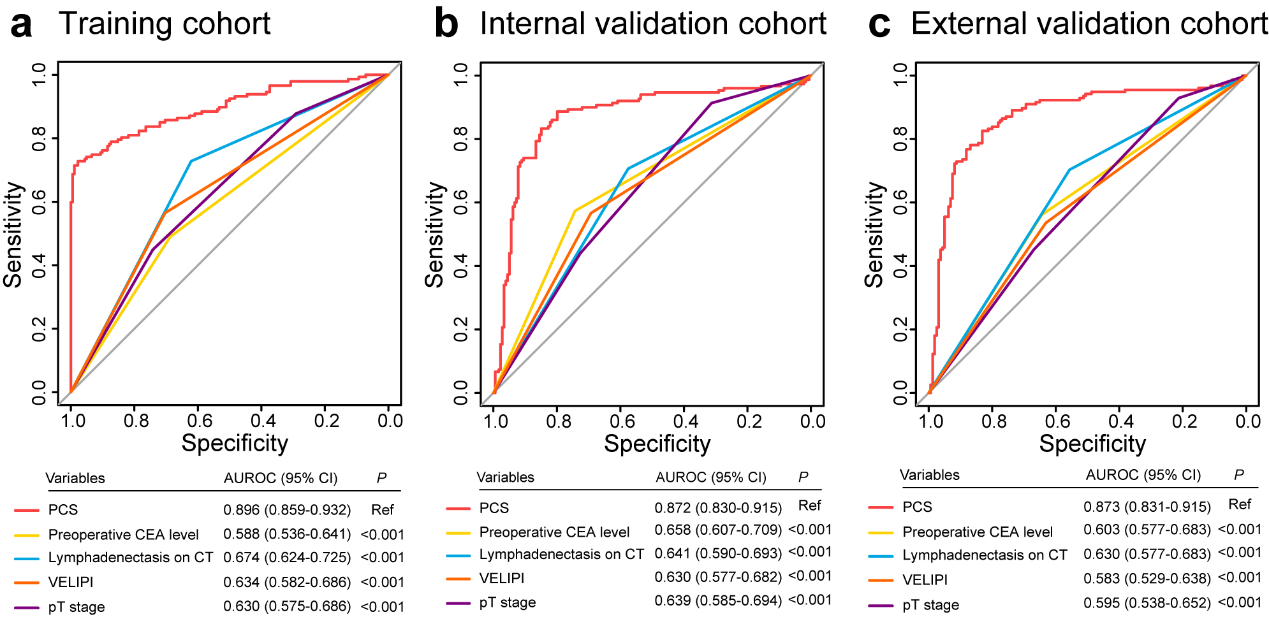


## Figure S4. ROC curves of the pathomics-collagen signature and other predictors.

The ROC curves of the pathomics-collagen signature and other predictors in the training cohort **(a)** and internal **(b)** and external **(c)** validation cohorts. The pathomics-collagen signature indicated significantly better discrimination than other predictors. Different variables are represented by different colors. *Abbreviations:* VELIPI, venous emboli and/or lymphatic invasion, and/or perineural invasion.


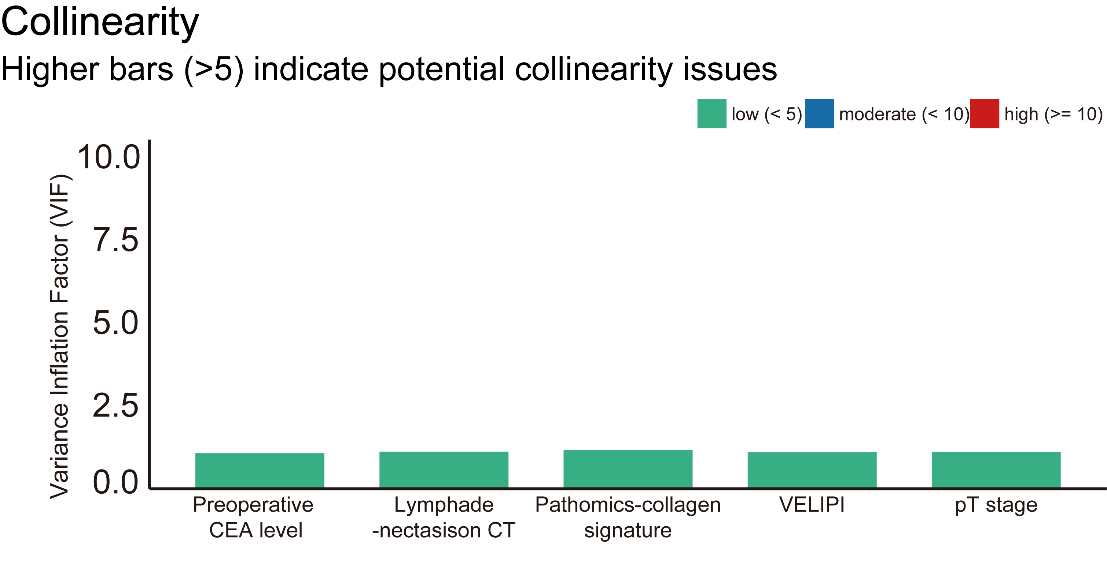


## Figure S5. Multicollinearity of the predictors of the PCS-nomogram.

The variance inflation factor of each predictor was less than 10; thus, there was no multicollinearity among these predictors. *Abbreviation:* VELIPI, venous emboli and/or lymphatic invasion and/or perineural invasion.


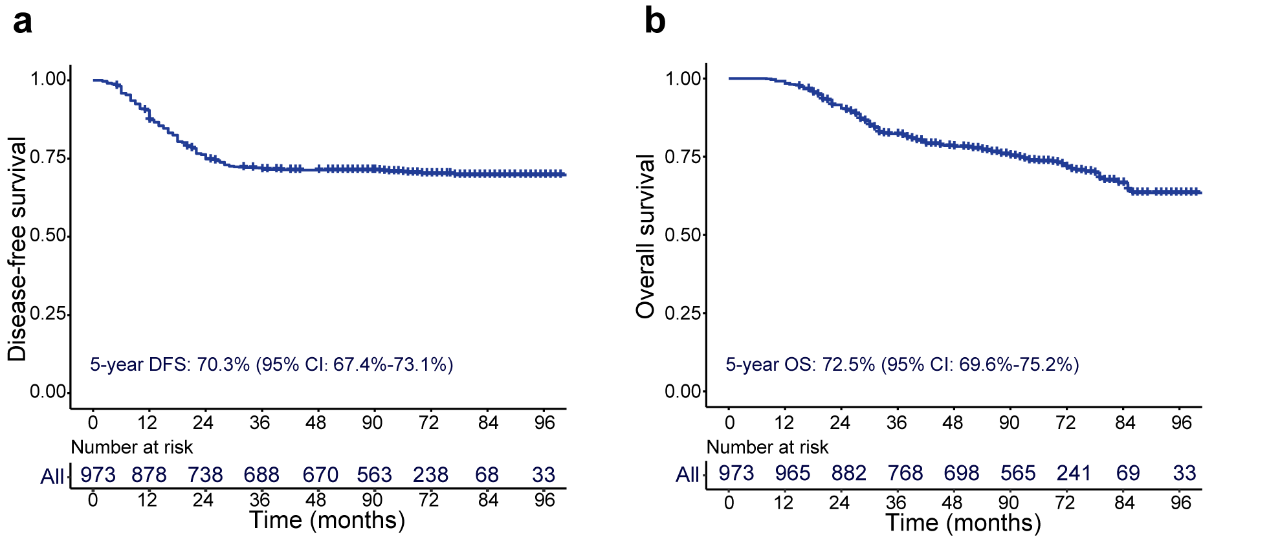


## Figure S6. Kaplan−Meier survival analysis in all patients.

**(a**) Disease-free survival status and **(b)** overall survival status of all patients.


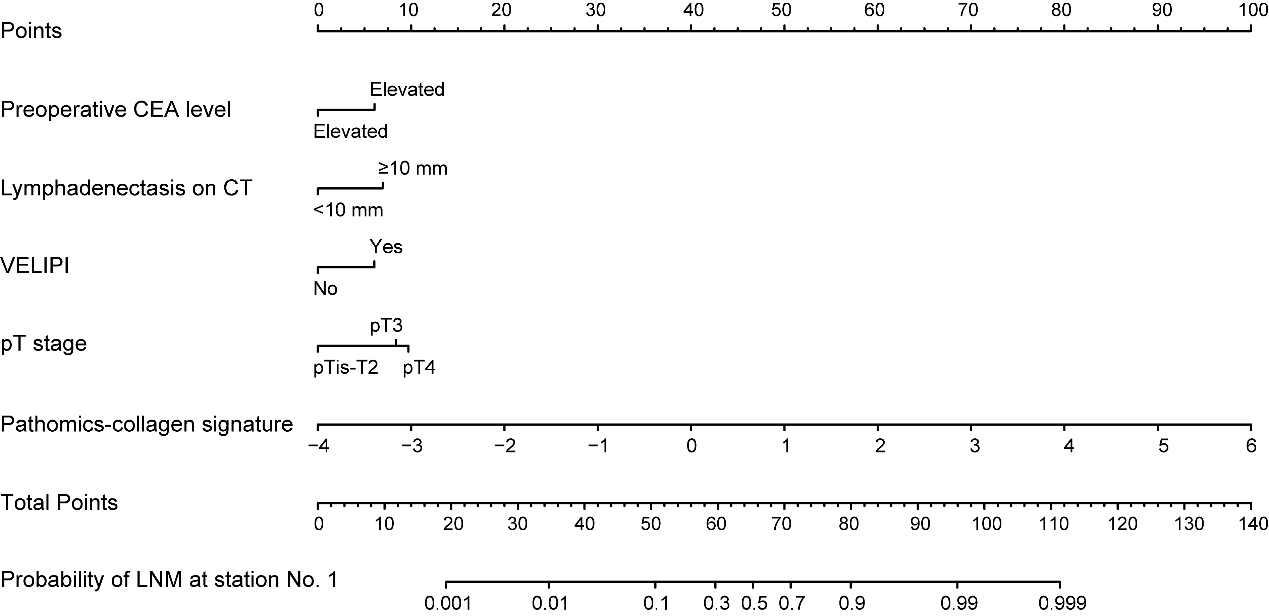


## Figure S7. PCS-nomogram to predict LNM at station No. 1 in patients with CRC.

The nomogram indicates the probability of LNM at station No. 1 in patients with CRC. For clinical use, an individual value is located on each variable axis, and a line is drawn upward to determine the number of points received for each variable value. The points of each covariate are added, and the total point value is located on the total points axis. Finally, a line is drawn straight down to the probability of LNM at station No. 1 to obtain the probability. *Abbreviations:* CRC, colorectal cancer; VELIPI, venous emboli and/or lymphatic invasion and/or perineural invasion; LNM, lymph node metastasis.


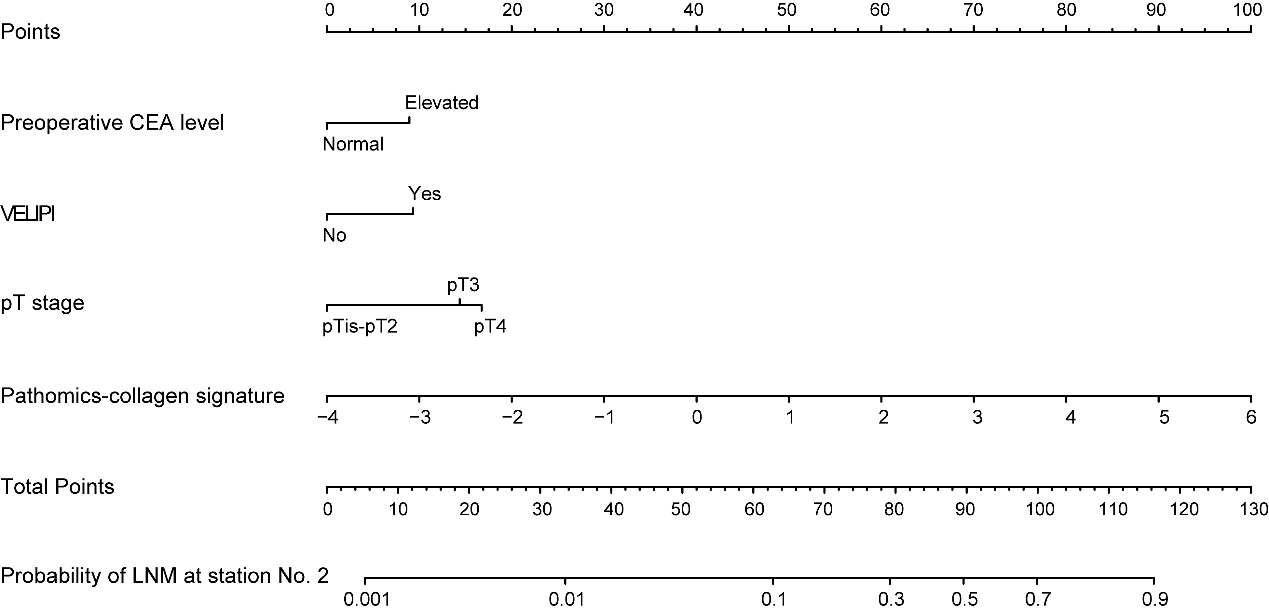


## Figure S8. PCS-nomogram to predict LNM at station No. 2 in patients with CRC.

The nomogram indicates the probability of LNM at station No. 2 in patients with CRC. For clinical use, an individual value is located on each variable axis, and a line is drawn upward to determine the number of points received for each variable value. The points of each covariate are added, and the total point value is located on the total points axis. Finally, a line is drawn straight down to the probability of LNM at station No. 2 to obtain the probability. *Abbreviations:* CRC, colorectal cancer; VELIPI, venous emboli and/or lymphatic invasion and/or perineural invasion; LNM, lymph node metastasis.


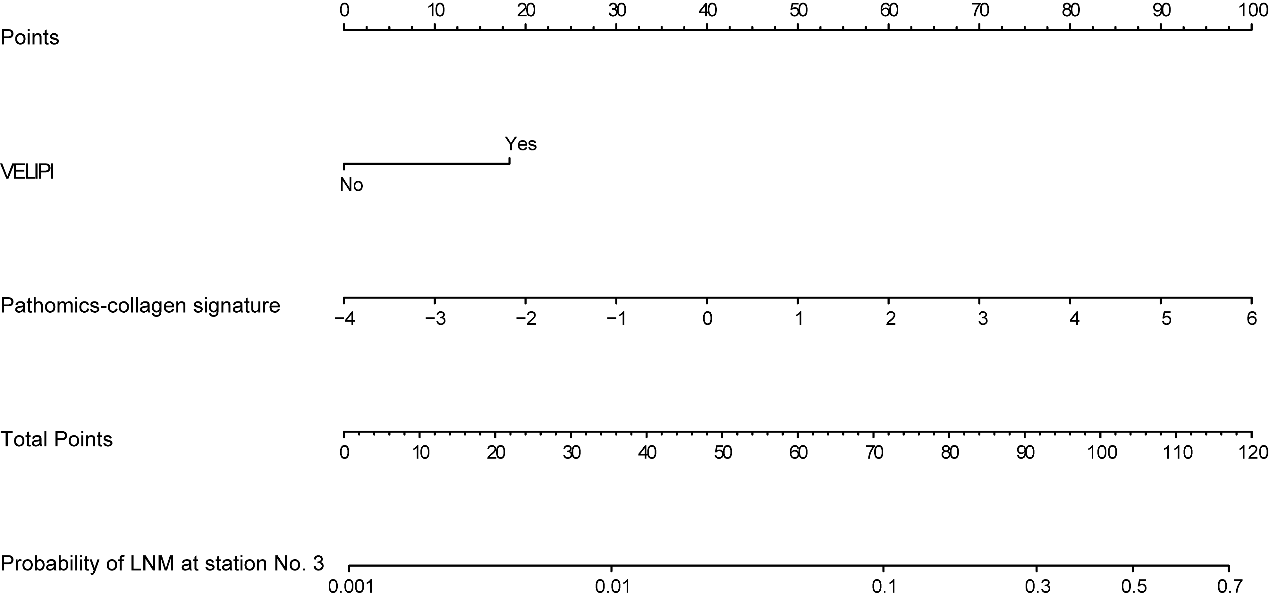


## Figure S9. PCS-nomogram to predict LNM at station No. 3 in patients with CRC.

The nomogram indicates the probability of LNM at station No. 3 in patients with CRC. For clinical use, an individual value is located on each variable axis, and a line is drawn upward to determine the number of points received for each variable value. The points of each covariate are added, and the total point value is located on the total points axis. Finally, a line is drawn straight down to the probability of LNM at station No. 3 to obtain the probability. *Abbreviations:* CRC, colorectal cancer; VELIPI, venous emboli and/or lymphatic invasion and/or perineural invasion; LNM, lymph node metastasis.


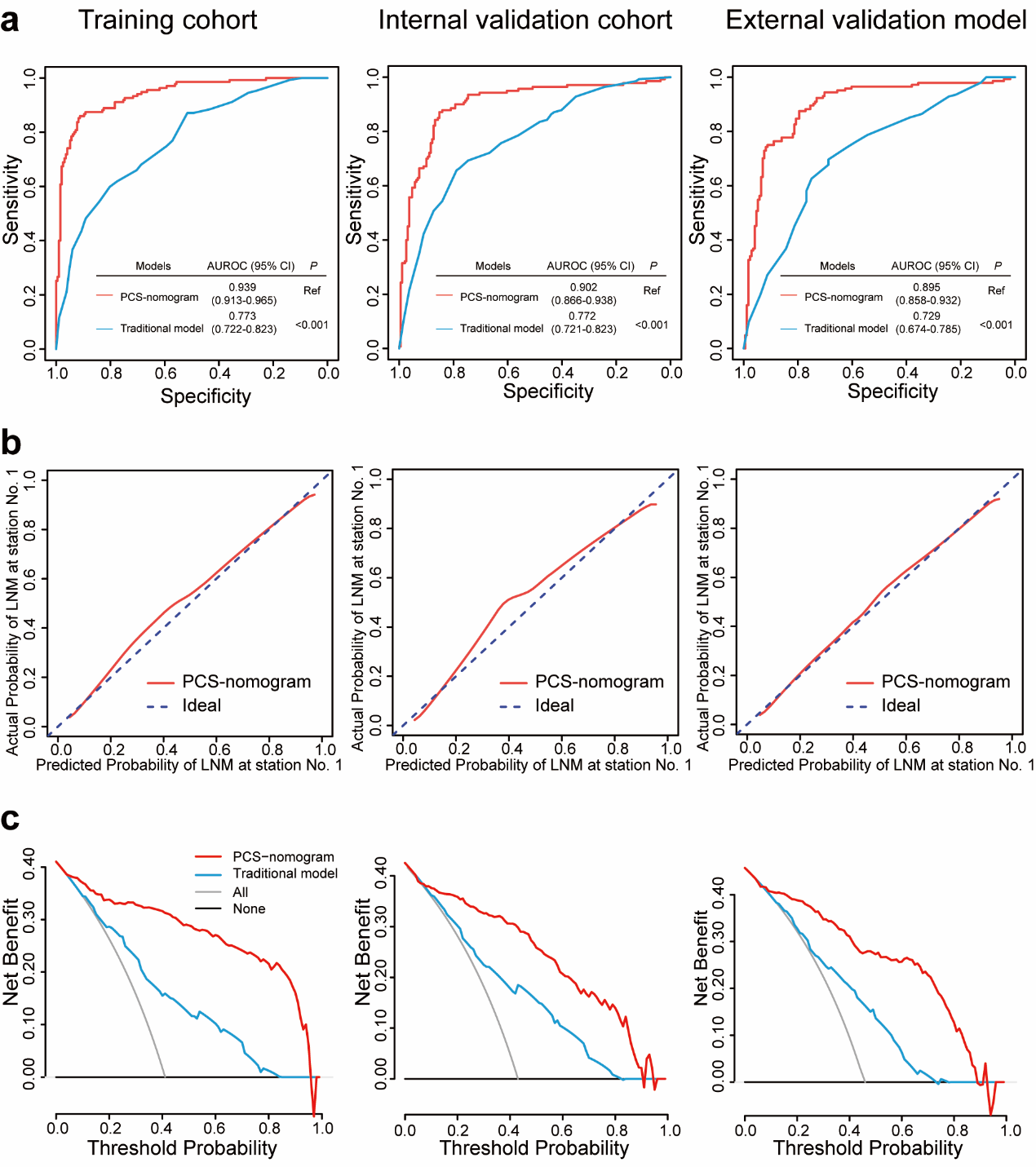


## Figure S10. Performance of the PCS-nomogram to predict LNM at station No. 1.

**(a)** The ROC curves of the PCS-nomogram and the traditional model to predict LNM at station No. 1 in the training cohort and the internal and external validation cohorts. **(b)** The calibration curves of the PCS-nomogram in the training cohort and the internal and external validation cohorts. **(c)** Decision curve analysis for the PCS-nomogram and the traditional model in each cohort. *Abbreviations:* PCS, pathomics-collagen signature; LNM, lymph node metastasis; AUROC, area under the receiver operating characteristic curve.


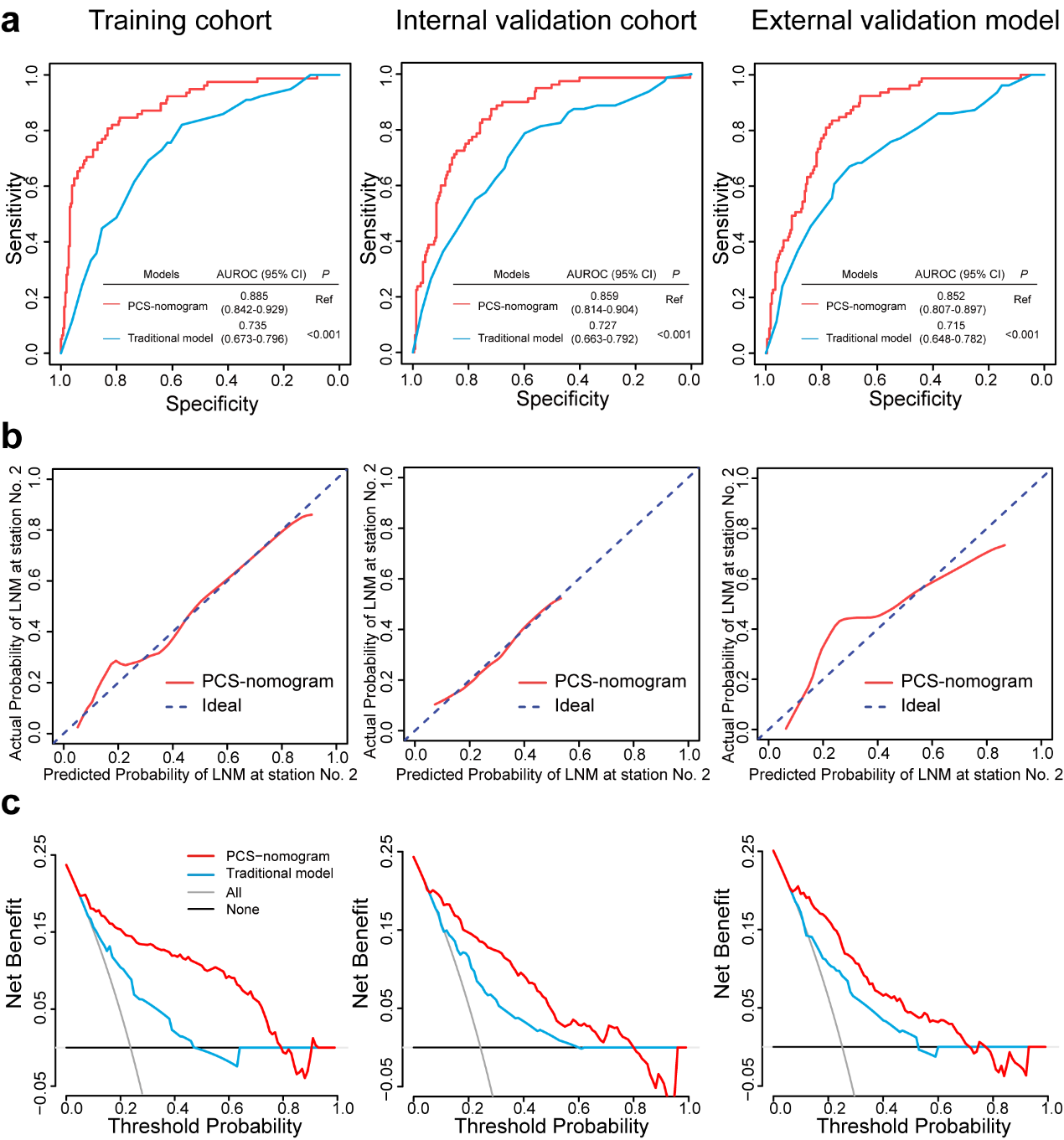


## Figure S11. Performance of the PCS-nomogram to predict LNM at station No. 2.

**(a)** The ROC curves of the PCS-nomogram and the traditional model to predict LNM at station No. 2 in the training cohort and the internal and external validation cohorts. **(b)** The calibration curves of the PCS-nomogram in the training cohort and the internal and external validation cohorts. **(c)** Decision curve analysis for the PCS-nomogram and the traditional model in each cohort. *Abbreviations:* PCS, pathomics-collagen signature; LNM, lymph node metastasis; AUROC, area under the receiver operating characteristic curve.


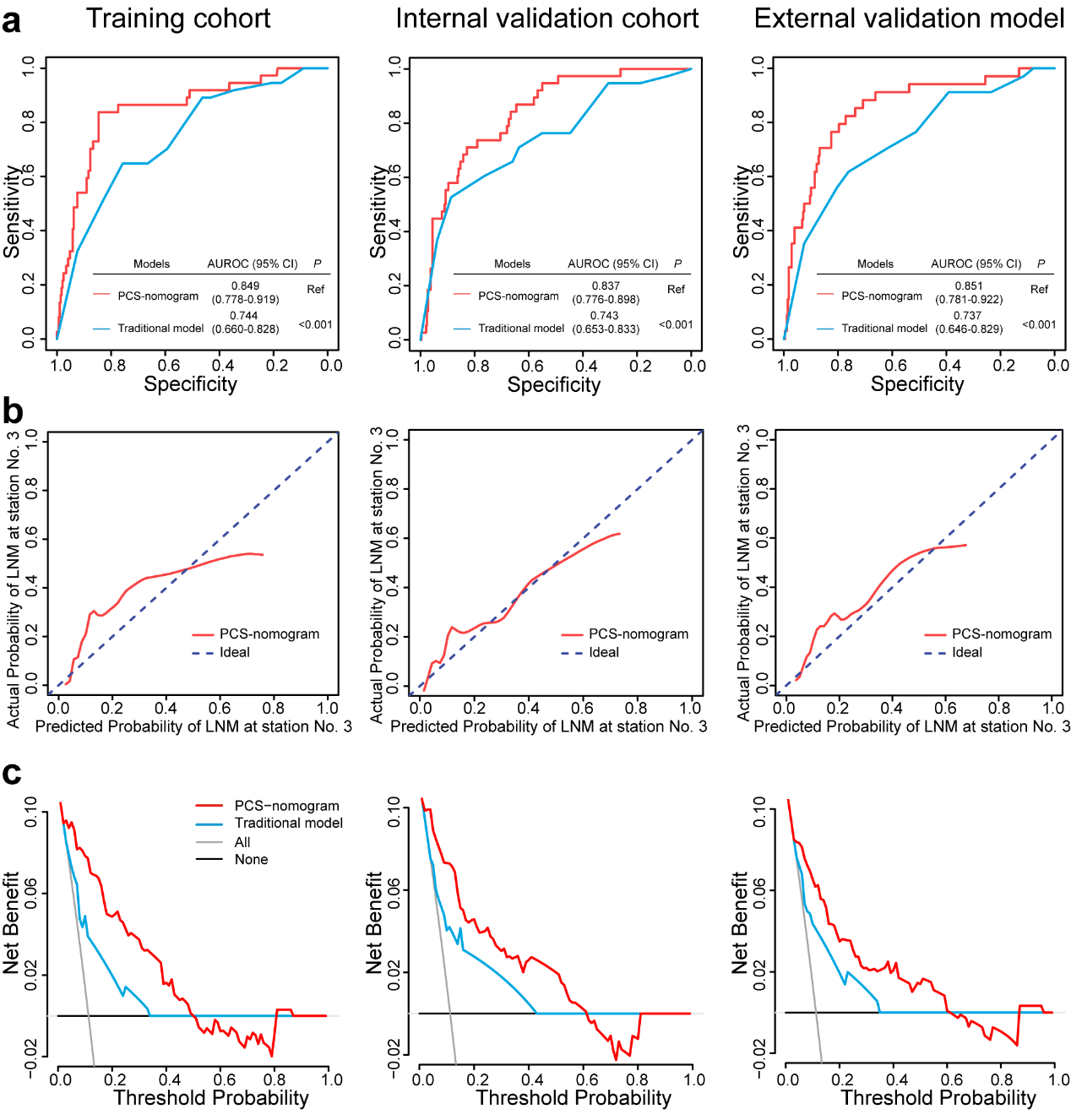


## Figure S12. Performance of the PCS-nomogram to predict LNM at station No. 3.

**(a)** The ROC curves of the PCS-nomogram and the traditional model to predict LNM at station No. 3 in the training cohort and the internal and external validation cohorts. **(b)** The calibration curves of the PCS-nomogram in the training cohort and the internal and external validation cohorts. **(c)** Decision curve analysis for the PCS-nomogram and the traditional model in each cohort. *Abbreviations:* PCS, pathomics-collagen signature; LNM, lymph node metastasis; AUROC, area under the receiver operating characteristic curve.

# IV. Supplementary Tables

## Table S1. Extracted 114 pathomics features.

| **No.** | **Pathomics feature characterization** |
| --- | --- |
| **Image quality features** | |
| 1-57 | IntegratedIntensity_α |
|  | MeanIntensity_α |
|  | StdIntensity_α |
|  | MinIntensity_α |
|  | MaxIntensity_α |
|  | IntegratedIntensityEdge_α |
|  | MeanIntensityEdge_α |
|  | StdIntensityEdge_α |
|  | MinIntensityEdge_α |
|  | MaxIntensityEdge_α |
|  | MassDisplacement_α |
|  | LowerQuartileIntensity_α |
|  | MedianIntensity_α |
|  | MADIntensity_α |
|  | UpperQuartileIntensity_α |
|  | CenterMassIntensity_X_α |
|  | CenterMassIntensity_Y_α |
|  | MaxIntensity_X_α |
|  | MaxIntensity _Y_α |
| **Image colocalization features** | |
| 58-66 | Correlation_Eosin_Haematoxylin |
|  | Costes_Eosin_Haematoxylin |
|  | Costes_Haematoxylin_Eosin |
|  | Manders_Eosin_Haematoxylin |
|  | Manders_Haematoxylin_Eosin |
|  | Overlap_Eosin_Haematoxylin |
|  | RWC_Eosin_Haematoxylin |
|  | RWC_Haematoxylin_Eosin |
|  | Slope_Eosin_Haematoxylin |
| **Image granularity features** | |
| 67-114 | Granularity_α_β |

α represents the type of images, which could be hematoxylin, eosin, and HE;

β represents the granular spectrum, which could be 1, 2, 3, 4, …, 15, 16.

*Abbreviation:* HE, hematoxylin and eosin.

## Table S2. Extracted 142 collagen features.

| **No.** | **Collagen feature characterization** |
| --- | --- |
| **Morphological features** | |
| 1 | Collagen area |
| 2 | Collagen number |
| 3 | Collagen length |
| 4 | Collagen width |
| 5 | Collagen straightness |
| 6 | Collagen crosslink density |
| 7 | Collagen crosslink space |
| 8 | Collagen orientation |
| **Texture features** | |
| 9–14 | mean, variance, skewness, kurtosis, energy, entropy of histogram |
| 15–95 | Contrast, correlation, energy and homogeneity of the Gray-Level Co-occurrence Matrix (GLCM) with four angles and five displacements |
| 96–142 | Mean and variance of the Gabor filter at four scales with six orientations |

## Table S3. Stratified analysis of the association between the PCS and LNM in the training, internal validation, and external validation cohorts.

| **Variables** | **AUROC (95% CI)** | | |
| --- | --- | --- | --- |
|  | **Training cohort** | **Internal validation cohort** | **External validation cohort** |
| **Age** |  |  |  |
| <60 | 0.898 (0.849, 0.948) | 0.871 (0.814, 0.928) | 0.856 (0.796, 0.916) |
| ≥60 | 0.894 (0.839, 0.949) | 0.876 (0.814, 0.938) | 0.893 (0.835, 0.951) |
| **Sex** |  |  |  |
| Male | 0.910 (0.868, 0.952) | 0.874 (0.819, 0.929) | 0.884 (0.832, 0.935) |
| Female | 0.877 (0.813, 0.941) | 0.868 (0.802, 0.935) | 0.863 (0.792, 0.934) |
| **Primary tumor location** |  |  |  |
| Left-sided | 0.907 (0.866, 0.948) | 0.868 (0.814, 0.923) | 0.869 (0.817, 0.921) |
| Right-sided | 0.873 (0.800, 0.947) | 0.881 (0.814, 0.948) | 0.887 (0.815, 0.955) |
| **Preoperative CEA level** |  |  |  |
| Normal | 0.927 (0.888, 0.966) | 0.863 (0.798, 0.927) | 0.830 (0.760, 0.899) |
| Elevated | 0.859 (0.795, 0.923) | 0.868 (0.795, 0.940) | 0.917 (0.869, 0.966) |
| **Preoperative CA19-9 level** |  |  |  |
| Normal | 0.908 (0.868, 0.947) | 0.875 (0.828, 0.922) | 0.872 (0.823, 0.922) |
| Elevated | 0.878 (0.799, 0.958) | 0.876 (0.788, 0.964) | 0.888 (0.816, 0.959) |
| **Lymphadenectasis on CT** |  |  |  |
| <10 mm | 0.916 (0.857, 0.975) | 0.855 (0.785, 0.925) | 0.808 (0.712, 0.892) |
| ≥10 mm | 0.884 (0.836, 0.932) | 0.880 (0.824, 0.937) | 0.919 (0.877, 0.962) |
| **Tumor differentiation** |  |  |  |
| Well or moderately | 0.916 (0.879, 0.952) | 0.867 (0.818, 0.915) | 0.865 (0.815, 0.916) |
| Poorly or undifferentiated | 0.817 (0.710, 0.924) | 0.885 (0.792, 0.977) | 0.892 (0.814, 0.970) |
| **VELIPI** |  |  |  |
| No | 0.893 (0.839, 0.948) | 0.850 (0.784, 0.916) | 0.869 (0.811, 0.928) |
| Yes | 0.888 (0.835, 0.942) | 0.883 (0.819, 0.948) | 0.885 (0.826, 0.944) |
| **Tumor size, cm** |  |  |  |
| <4 | 0.870 (0.806, 0.935) | 0.870 (0.808, 0.933) | 0.886 (0.825, 0.947) |
| ≥4 | 0.916 (0.874, 0.957) | 0.875 (0.818, 0.932) | 0.862 (0.803, 0.920) |
| **pT stage** |  |  |  |
| pTis-T2 | 0.865 (0.735, 0.994) | 0.860 (0.747, 0.973) | 0.912 (0.761, 1.000) |
| pT3 | 0.894 (0.839, 0.949) | 0.919 (0.869, 0.970) | 0.856 (0.791, 0.920) |
| pT4 | 0.913 (0.861, 0.965) | 0.839 (0.761, 0.916) | 0.878 (0.809, 0.946) |

*Abbreviations*: PCS, pathomics-collagen signature; LNM, lymph node metastasis; AUROC, area under the receiver operating characteristic curve; CI, confidence interval; CEA, carcinoembryonic antigen; CA19-9, carbohydrate antigen199; VELIPI, venous emboli and/or lymphatic invasion and/or perineural invasion.

## Table S4. Univariate and multivariable analyses of the predictors of LNM without the pathomics-collagen signature in the training cohort.

| **Variables** | **Univariate analysis** | | **Multivariable analysis** | |
| --- | --- | --- | --- | --- |
|  | **OR (95% CI)** | ***P*** | **OR (95% CI)** | ***P*** |
| **Age** | 0.985 (0.968, 1.003) | 0.104 |  |  |
| **Sex** |  |  |  |  |
| Male | Ref |  |  |  |
| Female | 1.023 (0.659, 1.587) | 0.920 |  |  |
| **Primary tumor location** |  |  |  |  |
| Left-sided | Ref |  |  |  |
| Right-sided | 1.042 (0.656, 1.655) | 0.860 |  |  |
| **Preoperative CEA level** |  |  |  |  |
| Normal | Ref |  | Ref |  |
| Elevated | 2.105 (1.342, 3.302) | 0.001 | 1.783 (1.071, 2.970) | 0.026 |
| **Preoperative CA19-9 level** |  |  |  |  |
| Normal | Ref |  |  |  |
| Elevated | 1.417 (0.851, 2.358) | 0.180 |  |  |
| **Lymphadenectasis on CT** |  |  |  |  |
| <10 mm | Ref |  | Ref |  |
| ≥10 mm | 4.381 (2.736, 7.015) | <0.001 | 4.579 (2.741, 7.650) | <0.001 |
| **Tumor differentiation** |  |  |  |  |
| Well or moderately | Ref |  |  |  |
| Poorly or undifferentiated | 1.583 (0.918, 2.732) | 0.099 | NA | NA |
| **VELIPI** |  |  |  |  |
| No | Ref |  | Ref |  |
| Yes | 3.074 (1.949, 4.848) | <0.001 | 2.788 (1.676, 4.637) | <0.001 |
| **Tumor size, cm** |  |  |  |  |
| <4 | Ref |  |  |  |
| ≥4 | 1.182 (0.762, 1.833) | 0.455 |  |  |
| **pT stage** |  |  |  |  |
| pTis-T2 | Ref |  | Ref |  |
| pT3 | 2.262 (1.208, 4.237) | 0.011 | 2.017 (1.019, 3.993) | 0.044 |
| pT4 | 4.135 (2.153, 7.941) | <0.001 | 3.807 (1.852, 7.825) | <0.001 |

*Abbreviations*: OR, odds ratio; CI, confidence interval; NA, not available; CEA, carcinoembryonic antigen; CA19-9, carbohydrate antigen199; VELIPI, venous emboli and/or lymphatic invasion and/or perineural invasion; Ref, reference.

## Table S5. NRI and IDI test for the prediction of LNM improvements of the PCS-nomogram compared with the traditional model.

| **Models** | **NRI (95% CI)** | ***P*** | **IDI (95% CI)** | ***P*** |
| --- | --- | --- | --- | --- |
| ***PCS-nomogram* vs*. Traditional model*** | | | | |
| Training cohort | 0.358 (0.255, 0.461) | <0.001 | 0.398 (0.341, 0.454) | <0.001 |
| Internal validation cohort | 0.216 (0.123, 0.309) | <0.001 | 0.214 (0.173, 0.255) | <0.001 |
| External validation cohort | 0.253 (0.144, 0.362) | <0.001 | 0.310 (0.262, 0.358) | <0.001 |

*Abbreviations*: CI, confidence interval; PCS, pathomics-collagen signature; LNM, lymph node metastasis; NRI, net reclassification improvement; IDI, integrated discrimination improvement.

**Table S6. Cox regression analysis of the predictors of survival in all patients.**

| **Variables** | **Univariate analysis** | ***P*** | **Multivariable analysis** | ***P*** |
| --- | --- | --- | --- | --- |
|  | **HR (95% CI)** |  | **HR (95% CI)** |  |
| ***Disease-free survival*** | | | | |
| Age (years) (≥60 vs. <60) | 0.999 (0.990, 1.008) | 0.873 |  |  |
| Sex (Male vs. Female) | 1.023 (0.808, 1.295) | 0.851 |  |  |
| Primary tumor location (Left-sided vs. Right-sided) | 0.874 (0.686, 1.113) | 0.274 |  |  |
| Pretreatment CEA (Elevated vs. Normal) | 1.470 (1.165, 1.854) | 0.001 | NA | NA |
| Pretreatment CA19-9 (Elevated vs. Normal) | 1.431 (1.113, 1.838) | 0.005 | 1.378 (1.072, 1.773) | 0.012 |
| Lymphadenectasis on CT (≥10 mm vs. <10 mm) | 1.621 (1.271, 2.067) | <0.001 | NA | NA |
| Tumor differentiation (Poorly or undifferentiated vs. Well or moderately) | 1.166 (0.885, 1.535) | 0.275 |  |  |
| VELIPI (Yes vs. No) | 1.763 (1.396, 2.226) | <0.001 | 1.335 (1.047, 1.704) | 0.020 |
| Tumor size, cm (≥4 vs. <4) | 1.071 (0.846, 1.354) | 0.571 |  |  |
| pT stage |  |  |  |  |
| pT3 vs. Tis-T2 | 2.365 (1.564, 3.576) | <0.001 | 1.745 (1.142, 2.666) | 0.010 |
| pT4 vs. Tis-T2 | 2.774 (1.829, 4.207) | <0.001 | 1.881 (1.222, 2.896) | 0.004 |
| Nomogram-predicted LNM probability (High vs. Low) | 2.872 (2.238, 3.686) | <0.001 | 2.328 (1.780, 3.045) | <0.001 |
| ***Overall survival*** | | | | |
| Age (years) (≥60 vs. <60) | 1.003 (0.994, 1.013) | 0.486 |  |  |
| Sex (Male vs. Female) | 1.014 (0.793, 1.298) | 0.909 |  |  |
| Primary tumor location (Left-sided vs. Right-sided) | 0.914 (0.708, 1.180) | 0.490 |  |  |
| Pretreatment CEA (Elevated vs. Normal) | 1.442 (1.131, 1.838) | 0.003 | NA | NA |
| Pretreatment CA19-9 (Elevated vs. Normal) | 1.453 (1.117, 1.891) | 0.005 | 1.401 (1.076, 1.825) | 0.012 |
| Lymphadenectasis on CT (≥10 mm vs. <10 mm) | 1.780 (1.376, 2.303) | <0.001 | NA | NA |
| Tumor differentiation (Poorly or undifferentiated vs. Well or moderately) | 1.034 (0.769, 1.390) | 0.825 |  |  |
| VELIPI (Yes vs. No) | 1.964 (1.537, 2.510) | <0.001 | 1.428 (1.106, 1.844) | 0.006 |
| Tumor size, cm (≥4 vs. <4) | 1.118 (0.873, 1.430) | 0.378 |  |  |
| pT stage |  |  |  |  |
| pT3 vs. Tis-T2 | 2.948 (1.837, 4.731) | <0.001 | 2.066 (1.274, 3.350) | 0.003 |
| pT4 vs. Tis-T2 | 3.843 (2.392, 6.175) | <0.001 | 2.459 (1.509, 4.007) | <0.001 |
| Nomogram-predicted LNM probability (High vs. Low) | 3.512 (2.679, 4.604) | <0.001 | 2.685 (2.011, 3.584) | <0.001 |

*Abbreviations*: HR, hazard ratio; CI, confidence interval; NA, not available; LNM, lymph node metastasis; VELIPI, venous emboli and/or lymphatic invasion and/or perineural invasion.

## Table S7. Univariate and multivariable analyses of the predictors of LNM at station No. 1 in the training cohort.

| **Variables** | **Univariate analysis** | | **Multivariable analysis** | |
| --- | --- | --- | --- | --- |
|  | **OR (95% CI)** | ***P*** | **OR (95% CI)** | ***P*** |
| **Age** | 0.984 (0.967, 1.002) | 0.089 | NA | NA |
| **Sex** |  |  |  |  |
| Male | Ref |  |  |  |
| Female | 1.083 (0.695, 1.688) | 0.725 |  |  |
| **Primary tumor location** |  |  |  |  |
| Left-sided | Ref |  |  |  |
| Right-sided | 1.164 (0.731, 1.856) | 0.522 |  |  |
| **Preoperative CEA level** |  |  |  |  |
| Normal | Ref |  | Ref |  |
| Elevated | 2.335 (1.482, 3.678) | <0.001 | 2.487 (1.221, 5.066) | 0.012 |
| **Preoperative CA19-9 level** |  |  |  |  |
| Normal | Ref |  |  |  |
| Elevated | 1.508 (0.904, 2.516) | 0.115 |  |  |
| **Lymphadenectasis on CT** |  |  |  |  |
| <10 mm | Ref |  | Ref |  |
| ≥10 mm | 3.939 (2.450, 6.332) | <0.001 | 2.838 (1.389, 5.802) | 0.004 |
| **Tumor differentiation** |  |  |  |  |
| Well or moderately | Ref |  |  |  |
| Poorly or undifferentiated | 1.638 (0.949, 2.827) | 0.076 | NA | NA |
| **VELIPI** |  |  |  |  |
| No | Ref |  | Ref |  |
| Yes | 3.131 (1.980, 4.952) | <0.001 | 2.480 (1.231, 4.996) | 0.011 |
| **Tumor size, cm** |  |  |  |  |
| <4 | Ref |  |  |  |
| ≥4 | 1.113 (0.714, 1.734) | 0.637 |  |  |
| **pT stage** |  |  |  |  |
| pTis-T2 | Ref |  | Ref |  |
| pT3 | 2.489 (1.287, 4.814) | 0.007 | 3.505 (1.227, 10.011) | 0.019 |
| pT4 | 4.539 (2.300, 8.956) | <0.001 | 4.283 (1.435, 12.779) | 0.009 |
| **PCS** | 4.747 (3.245, 6.943) | <0.001 | 4.479 (2.986, 6.719) | <0.001 |

*Abbreviations*: OR, odds ratio; CI, confidence interval; NA, not available; CEA, carcinoembryonic antigen; CA19-9, carbohydrate antigen199; VELIPI, venous emboli and/or lymphatic invasion and/or perineural invasion; Ref, reference; PCS, pathomics-collagen signature.

## Table S8. Univariate and multivariable analyses of the predictors of LNM at station No. 2 in the training cohort.

| **Variables** | **Univariate analysis** | | **Multivariable analysis** | |
| --- | --- | --- | --- | --- |
|  | **OR (95% CI)** | ***P*** | **OR (95% CI)** | ***P*** |
| **Age** | 1.000 (0.979, 1.012) | 0.975 |  |  |
| **Sex** |  |  |  |  |
| Male | Ref |  |  |  |
| Female | 1.057 (0.633, 1.765) | 0.832 |  |  |
| **Primary tumor location** |  |  |  |  |
| Left-sided | Ref |  |  |  |
| Right-sided | 1.030 (0.601, 1.768) | 0.913 |  |  |
| **Preoperative CEA level** |  |  |  |  |
| Normal | Ref |  | Ref |  |
| Elevated | 2.199 (1.313, 3.683) | 0.003 | 2.080 (1.065, 4.062) | 0.032 |
| **Preoperative CA19-9 level** |  |  |  |  |
| Normal | Ref |  |  |  |
| Elevated | 1.152 (0.626, 2.120) | 0.649 |  |  |
| **Lymphadenectasis on CT** |  |  |  |  |
| <10 mm | Ref |  |  |  |
| ≥10 mm | 2.206 (1.291, 3.771) | 0.004 | NA | NA |
| **Tumor differentiation** |  |  |  |  |
| Well or moderately | Ref |  |  |  |
| Poorly or undifferentiated | 1.302 (0.704, 2.408) | 0.400 |  |  |
| **VELIPI** |  |  |  |  |
| No | Ref |  | Ref |  |
| Yes | 2.712 (1.610, 4.570) | <0.001 | 2.149 (1.091, 4.235) | 0.027 |
| **Tumor size, cm** |  |  |  |  |
| <4 | Ref |  |  |  |
| ≥4 | 1.534 (0.908, 2.594) | 0.110 |  |  |
| **pT stage** |  |  |  |  |
| pTis-T2 | Ref |  | Ref |  |
| pT3 | 3.192 (1.270, 8.025) | 0.014 | 3.248 (1.074, 9.825) | 0.037 |
| pT4 | 5.709 (2.271, 14.353) | <0.001 | 3.947 (1.303, 11.956) | 0.015 |
| **PCS** | 2.316 (1.931, 2.776) | <0.001 | 2.271 (1.880, 2.745) | <0.001 |

*Abbreviations*: OR, odds ratio; CI, confidence interval; NA, not available; CEA, carcinoembryonic antigen; CA19-9, carbohydrate antigen199; VELIPI, venous emboli and/or lymphatic invasion and/or perineural invasion; Ref, reference; PCS, pathomics-collagen signature.

## Table S9. Univariate and multivariable analyses of the predictors of LNM at station No. 3 in the training cohort.

| **Variables** | **Univariate analysis** | | **Multivariable analysis** | |
| --- | --- | --- | --- | --- |
|  | **OR (95% CI)** | ***P*** | **OR (95% CI)** | ***P*** |
| **Age** | 0.991 (0.964, 1.018) | 0.509 |  |  |
| **Sex** |  |  |  |  |
| Male | Ref |  |  |  |
| Female | 0.615 (0.298, 1.271) | 0.189 |  |  |
| **Primary tumor location** |  |  |  |  |
| Left-sided | Ref |  |  |  |
| Right-sided | 1.459 (0.724, 2.941) | 0.291 |  |  |
| **Preoperative CEA level** |  |  |  |  |
| Normal | Ref |  |  |  |
| Elevated | 2.236 (1.119, 4.469) | 0.023 | NA | NA |
| **Preoperative CA19-9 level** |  |  |  |  |
| Normal | Ref |  |  |  |
| Elevated | 1.143 (0.500, 2.615) | 0.752 |  |  |
| **Lymphadenectasis on CT** |  |  |  |  |
| <10 mm | Ref |  |  |  |
| ≥10 mm | 3.027 (1.380, 6.638) | 0.006 | NA | NA |
| **Tumor differentiation** |  |  |  |  |
| Well or moderately | Ref |  |  |  |
| Poorly or undifferentiated | 1.137 (0.494, 2.620) | 0.762 |  |  |
| **VELIPI** |  |  |  |  |
| No | Ref |  | Ref |  |
| Yes | 3.854 (1.832, 8.107) | <0.001 | 3.371 (1.487, 7.642) | 0.004 |
| **Tumor size, cm** |  |  |  |  |
| <4 | Ref |  |  |  |
| ≥4 | 1.318 (0.652, 2.664) | 0.441 |  |  |
| **pT stage** |  |  |  |  |
| pTis-T2 | Ref |  |  |  |
| pT3 | 4.582 (1.028, 20.415) | 0.046 | NA | NA |
| pT4 | 6.537 (1.468, 29.103) | 0.014 | NA | NA |
| **PCS** | 1.955 (1.597, 2.394) | <0.001 | 1.948 (1.576, 2.407) | <0.001 |

*Abbreviations*: OR, odds ratio; CI, confidence interval; NA, not available; CEA, carcinoembryonic antigen; CA19-9, carbohydrate antigen199; VELIPI, venous emboli and/or lymphatic invasion and/or perineural invasion; Ref, reference; PCS, pathomics-collagen signature.

## Table S10. Predictive power of LNM at station No. 1 between the PCS-nomogram and traditional model.

| **Variables** | **AUROC** | **Sensitivity** | **Specificity** | **Accuracy** | **PPV** | **NPV** |
| --- | --- | --- | --- | --- | --- | --- |
| **Training cohort** | | | | | | |
| PCS-nomogram | 0.939  (0.913, 0.965) | 85.9%  (79.1%, 90.8%) | 91.2%  (86.4%, 94.5%) | 89.1%  (85.2%, 92.0%) | 88.2%  (80.5%, 91.9%) | 90.3%  (85.4%, 93.7%) |
| Traditional model | 0.773  (0.722, 0.823) | 88.9%  (83.5%, 93.1%) | 49.7%  (42.7%, 56.8%) | 66.0%  (60.6%, 70.9%) | 55.6%  (48.9%, 62.0%) | 86.4%  (78.7%, 91.6%) |
| **Internal validation cohort** | | | | | | |
| PCS-nomogram | 0.902  (0.866, 0.938) | 87.1%  (80.6%, 91.7%) | 85.2%  (79.4%, 89.5%) | 86.0%  (81.9%, 89.4%) | 81.3%  (74.3%, 86.8%) | 89.9%  (84.7%, 93.5%) |
| Traditional model | 0.772  (0.721, 0.823) | 65.7%  (57.5%, 73.1%) | 78.8%  (82.5%, 84.1%) | 73.3%  (68.2%, 77.7%) | 69.7%  (61.4%, 76.9%) | 75.6%  (69.2%, 81.1%) |
| **External validation cohort** | | | | | | |
| PCS-nomogram | 0.895  (0.858, 0.932) | 87.5%  (81.1%, 91.9%) | 79.5%  (72.9%, 84.9%) | 83.2%  (78.6%, 86.9%) | 78.3%  (71.3%, 83.9%) | 88.3%  (82.3%, 92.5%) |
| Traditional model | 0.729  (0.674, 0.785) | 70.8%  (62.9%, 77.6%) | 67.3%  (59.9%, 73.8%) | 68.9%  (63.6%, 73.7%) | 64.6%  (56.8%, 71.6%) | 73.2%  (65.8%, 79.6%) |

*Abbreviations*: LNM, lymph node metastasis; AUROC, area under the receiver operating characteristic curve; PCS, pathomics-collagen signature; PPV, positive predictive value; NPV, negative predictive value.

## Table S11. Predictive power of LNM at station No. 2 between the PCS-nomogram and traditional model.

| **Variables** | **AUROC** | **Sensitivity** | **Specificity** | **Accuracy** | **PPV** | **NPV** |
| --- | --- | --- | --- | --- | --- | --- |
| **Training cohort** | | | | | | |
| PCS-nomogram | 0.885  (0.842, 0.929) | 80.8%  (70.7%, 88.0%) | 88.3%  (78.2%, 87.4%) | 82.7%  (78.2%, 86.4%) | 52.5%  (43.6%, 61.2%) | 93.3%  (89.2%, 95.9%) |
| Traditional model | 0.735  (0.673, 0.796) | 82.1%  (72.1%, 89.9%) | 56.6%  (50.4%, 62.6%) | 62.6%  (57.3%, 67.7%) | 37.0%  (30.2%, 44.4%) | 91.0%  (85.5%, 94.6%) |
| **Internal validation cohort** | | | | | | |
| PCS-nomogram | 0.859  (0.814, 0.904) | 87.5%  (78.5%, 93.1%) | 71.9%  (66.0%, 77.1%) | 75.7%  (70.8%, 80.0%) | 50.0%  (41.8%, 58.2%) | 94.7%  (90.5%, 97.1%) |
| Traditional model | 0.727  (0.663, 0.792) | 78.8%  (68.6%, 86.3%) | 59.8%  (63.6%, 65.7%) | 64.4%  (59.1%, 69.4%) | 38.7%  (31.5%, 46.3%) | 89.8%  (84.2%, 93.5%) |
| **External validation cohort** | | | | | | |
| PCS-nomogram | 0.852  (0.807, 0.897) | 83.5%  (73.9%, 90.1%) | 76.3%  (70.5%, 81.3%) | 78.1%  (73.2%, 82.3%) | 54.1%  (45.3%, 62.7%) | 93.3%  (88.8%, 86.0%) |
| Traditional model | 0.715  (0.648, 0.782) | 67.1%  (56.1%, 76.4%) | 69.9%  (63.8%, 75.4%) | 69.2%  (63.9%, 74.0%) | 42.7%  (34.4%, 51.5%) | 86.4%  (80.8%, 90.5%) |

*Abbreviations*: LNM, lymph node metastasis; AUROC, area under the receiver operating characteristic curve; PCS, pathomics-collagen signature; PPV, positive predictive value; NPV, negative predictive value.

## Table S12. Predictive power of LNM at station No. 3 between the PCS-nomogram and traditional model.

| **Variables** | **AUROC** | **Sensitivity** | **Specificity** | **Accuracy** | **PPV** | **NPV** |
| --- | --- | --- | --- | --- | --- | --- |
| **Training cohort** | | | | | | |
| PCS-nomogram | 0.849  (0.778, 0.919) | 83.8%  (68.9%, 92.3%) | 84.6%  (80.0%, 88.3%) | 84.5%  (80.2%, 88.0%) | 40.3%  (30.0%, 51.4%) | 97.6%  (94.9%, 98.9%) |
| Traditional model | 0.744  (0.660, 0.828) | 64.9%  (48.8%, 78.2%) | 75.7%  (70.5%, 80.3%) | 74.5%  (69.5%, 78.9%) | 25.3%  (17.6%, 34.8%) | 94.4%  (90.7%, 96.7%) |
| **Internal validation cohort** | | | | | | |
| PCS-nomogram | 0.837  (0.776, 0.898) | 71.1%  (55.2%, 83.0%) | 82.8%  (78.1%, 86.7%) | 81.5%  (76.9%, 85.3%) | 35.6%  (25.3%, 46.2%) | 95.6%  (92.4%, 97.5%) |
| Traditional model | 0.743  (0.653, 0.833) | 60.5%  (44.7%, 74.4%) | 76.3%  (71.1%, 80.8%) | 74.5%  (69.5%, 78.9%) | 25.0%  (17.3%, 34.7%) | 93.7%  (17.3%, 34.7%) |
| **External validation cohort** | | | | | | |
| PCS-nomogram | 0.851  (0.781, 0.922) | 83.5%  (73.9%, 90.1%) | 76.3%  (70.5%, 81.3%) | 78.1%  (73.2%, 82.3) | 54.1%  (45.3%, 62.7%) | 93.3%  (88.8%, 96.0%) |
| Traditional model | 0.737  (0.646, 0.829) | 67.1%  (56.1%, 76.4%) | 69.9%  (63.8%, 75.4%) | 69.2%  (63.9%, 74.0%) | 42.7%  (34.4%, 51.5%) | 86.4%  (80.8%, 90.5%) |

*Abbreviations*: LNM, lymph node metastasis; AUROC, area under the receiver operating characteristic curve; PCS, pathomics-collagen signature; PPV, positive predictive value; NPV, negative predictive value.

## Table S13. Univariate and multivariable analyses of the predictors of LNM at station No. 1 without pathomics-collagen signature in the training cohort.

| **Variables** | **Univariate analysis** | | **Multivariable analysis** | |
| --- | --- | --- | --- | --- |
|  | **OR (95% CI)** | ***P*** | **OR (95% CI)** | ***P*** |
| **Age** | 0.984 (0.967, 1.002) | 0.089 | NA | NA |
| **Sex** |  |  |  |  |
| Male | Ref |  |  |  |
| Female | 1.083 (0.695, 1.688) | 0.725 |  |  |
| **Primary tumor location** |  |  |  |  |
| Left-sided | Ref |  |  |  |
| Right-sided | 1.164 (0.731, 1.856) | 0.522 |  |  |
| **Preoperative CEA level** |  |  |  |  |
| Normal | Ref |  | Ref |  |
| Elevated | 2.335 (1.482, 3.678) | <0.001 | 2.034 (1.220, 3.390) | 0.006 |
| **Preoperative CA19-9 level** |  |  |  |  |
| Normal | Ref |  |  |  |
| Elevated | 1.508 (0.904, 2.516) | 0.115 |  |  |
| **Lymphadenectasis on CT** |  |  |  |  |
| <10 mm | Ref |  | Ref |  |
| ≥10 mm | 3.939 (2.450, 6.332) | <0.001 | 3.998 (2.383, 6.706) | <0.001 |
| **Tumor differentiation** |  |  |  |  |
| Well or moderately | Ref |  |  |  |
| Poorly or undifferentiated | 1.638 (0.949, 2.827) | 0.076 | NA | NA |
| **VELIPI** |  |  |  |  |
| No | Ref |  | Ref |  |
| Yes | 3.131 (1.980, 4.952) | <0.001 | 2.777 (1.670, 4.618) | <0.001 |
| **Tumor size, cm** |  |  |  |  |
| <4 | Ref |  |  |  |
| ≥4 | 1.113 (0.714, 1.734) | 0.637 |  |  |
| **pT stage** |  |  |  |  |
| pTis-T2 | Ref |  | Ref |  |
| pT3 | 2.489 (1.287, 4.814) | 0.007 | 2.212 (1.088, 4.499) | 0.028 |
| pT4 | 4.539 (2.300, 8.956) | <0.001 | 4.118 (1.961, 8.645) | <0.001 |

*Abbreviations*: OR, odds ratio; CI, confidence interval; NA, not available; CEA, carcinoembryonic antigen; CA19-9, carbohydrate antigen199; VELIPI, venous emboli and/or lymphatic invasion and/or perineural invasion; Ref, reference.

## Table S14. Univariate and multivariable analyses of the predictors of LNM at station No. 2 without pathomics-collagen signature in the training cohort.

| **Variables** | **Univariate analysis** | | **Multivariable analysis** | |
| --- | --- | --- | --- | --- |
|  | **OR (95% CI)** | ***P*** | **OR (95% CI)** | ***P*** |
| **Age** | 1.000 (0.979, 1.012) | 0.975 |  |  |
| **Sex** |  |  |  |  |
| Male | Ref |  |  |  |
| Female | 1.057 (0.633, 1.765) | 0.832 |  |  |
| **Primary tumor location** |  |  |  |  |
| Left-sided | Ref |  |  |  |
| Right-sided | 1.030 (0.601, 1.768) | 0.913 |  |  |
| **Preoperative CEA level** |  |  |  |  |
| Normal | Ref |  | Ref |  |
| Elevated | 2.199 (1.313, 3.683) | 0.003 | 1.932 (1.115, 3.346) | 0.019 |
| **Preoperative CA19-9 level** |  |  |  |  |
| Normal | Ref |  |  |  |
| Elevated | 1.152 (0.626, 2.120) | 0.649 |  |  |
| **Lymphadenectasis on CT** |  |  |  |  |
| <10 mm | Ref |  | Ref |  |
| ≥10 mm | 2.206 (1.291, 3.771) | 0.004 | 1.948 (1.104, 3.435) | 0.021 |
| **Tumor differentiation** |  |  |  |  |
| Well or moderately | Ref |  |  |  |
| Poorly or undifferentiated | 1.302 (0.704, 2.408) | 0.400 |  |  |
| **VELIPI** |  |  |  |  |
| No | Ref |  | Ref |  |
| Yes | 2.712 (1.610, 4.570) | <0.001 | 2.183 (1.261, 3.778) | 0.005 |
| **Tumor size, cm** |  |  |  |  |
| <4 | Ref |  |  |  |
| ≥4 | 1.534 (0.908, 2.594) | 0.110 |  |  |
| **pT stage** |  |  |  |  |
| pTis-T2 | Ref |  | Ref |  |
| pT3 | 3.192 (1.270, 8.025) | 0.014 | 2.769 (1.080, 7.099) | 0.034 |
| pT4 | 5.709 (2.271, 14.353) | <0.001 | 4.736 (1.836, 12.212) | 0.001 |

*Abbreviations*: OR, odds ratio; CI, confidence interval; NA, not available; CEA, carcinoembryonic antigen; CA19-9, carbohydrate antigen199; VELIPI, venous emboli and/or lymphatic invasion and/or perineural invasion; Ref, reference; PCS, pathomics-collagen signature.

## Table S15. Univariate and multivariable analyses of the predictors of LNM at station No. 3 without pathomics-collagen signature in the training cohort.

| **Variables** | **Univariate analysis** | | **Multivariable analysis** | |
| --- | --- | --- | --- | --- |
|  | **OR (95% CI)** | ***P*** | **OR (95% CI)** | ***P*** |
| **Age** | 0.991 (0.964, 1.018) | 0.509 |  |  |
| **Sex** |  |  |  |  |
| Male | Ref |  |  |  |
| Female | 0.615 (0.298, 1.271) | 0.189 |  |  |
| **Primary tumor location** |  |  |  |  |
| Left-sided | Ref |  |  |  |
| Right-sided | 1.459 (0.724, 2.941) | 0.291 |  |  |
| **Preoperative CEA level** |  |  |  |  |
| Normal | Ref |  |  |  |
| Elevated | 2.236 (1.119, 4.469) | 0.023 | NA | NA |
| **Preoperative CA19-9 level** |  |  |  |  |
| Normal | Ref |  |  |  |
| Elevated | 1.143 (0.500, 2.615) | 0.752 |  |  |
| **Lymphadenectasis on CT** |  |  |  |  |
| <10 mm | Ref |  | Ref |  |
| ≥10 mm | 3.027 (1.380, 6.638) | 0.006 | 2.876 (1.287, 6.426) | 0.010 |
| **Tumor differentiation** |  |  |  |  |
| Well or moderately | Ref |  |  |  |
| Poorly or undifferentiated | 1.137 (0.494, 2.620) | 0.762 |  |  |
| **VELIPI** |  |  |  |  |
| No | Ref |  | Ref |  |
| Yes | 3.854 (1.832, 8.107) | <0.001 | 3.247 (1.513, 6.969) | 0.003 |
| **Tumor size, cm** |  |  |  |  |
| <4 | Ref |  |  |  |
| ≥4 | 1.318 (0.652, 2.664) | 0.441 |  |  |
| **pT stage** |  |  |  |  |
| pTis-T2 | Ref |  | Ref |  |
| pT3 | 4.582 (1.028, 20.415) | 0.046 | 3.756 (0.826, 17.084) | 0.087 |
| pT4 | 6.537 (1.468, 29.103) | 0.014 | 4.799 (1.049, 21.962) | 0.043 |

*Abbreviations*: OR, odds ratio; CI, confidence interval; NA, not available; CEA, carcinoembryonic antigen; CA19-9, carbohydrate antigen199; VELIPI, venous emboli and/or lymphatic invasion and/or perineural invasion; Ref, reference; PCS, pathomics-collagen signature.

## Table S16. NRI and IDI test for prediction of LNM at station No 1. improvements of PCS-nomogram compared with the traditional model.

| **Models** | **NRI (95% CI)** | ***P*** | **IDI (95% CI)** | ***P*** |
| --- | --- | --- | --- | --- |
| ***PCS-nomogram* vs*. Traditional model*** | | | | |
| Training cohort | 0.395 (0.292, 0.498) | <0.001 | 0.385 (0.327, 0.443) | <0.001 |
| Internal validation cohort | 0.328 (0.227, 0.428) | <0.001 | 0.262 (0.216, 0.307) | <0.001 |
| External validation cohort | 0.277 (0.164, 0.391) | <0.001 | 0.307 (0.285, 0.355) | <0.001 |

*Abbreviations*: CI, confidence interval; PCS, pathomics-collagen signature; LNM, lymph node metastasis; NRI, net reclassification improvement; IDI, integrated discrimination improvement.

## Table S17. NRI and IDI test for prediction of LNM at station No. 2 improvements of PCS-nomogram compared with the traditional model.

| **Models** | **NRI (95% CI)** | ***P*** | **IDI (95% CI)** | ***P*** |
| --- | --- | --- | --- | --- |
| ***PCS-nomogram* vs*. Traditional model*** | | | | |
| Training cohort | 0.270 (0.142, 0.399) | <0.001 | 0.299 (0.235, 0.364) | <0.001 |
| Internal validation cohort | 0.230 (0.127, 0.333) | <0.001 | 0.171 (0.122, 0.221) | <0.001 |
| External validation cohort | 0.262 (0.138, 0.387) | <0.001 | 0.179 (0.129, 0.230) | <0.001 |

*Abbreviations*: CI, confidence interval; PCS, pathomics-collagen signature; LNM, lymph node metastasis; NRI, net reclassification improvement; IDI, integrated discrimination improvement.

## Table S18. NRI and IDI test for prediction of LNM at station No. 3 improvements of PCS-nomogram compared with the traditional model.

| **Models** | **NRI (95% CI)** | ***P*** | **IDI (95% CI)** | ***P*** |
| --- | --- | --- | --- | --- |
| ***PCS-nomogram* vs*. Traditional model*** | | | | |
| Training cohort | 0.324 (0.147, 0.502) | <0.001 | 0.139 (0.076, 0.202) | <0.001 |
| Internal validation cohort | 0.167 (0.002, 0.390) | 0.048 | 0.056 (0.003, 0.108) | 0.037 |
| External validation cohort | 0.227 (0.004, 0.414) | 0.017 | 0.118 (0.056, 0.181) | <0.001 |

*Abbreviations*: CI, confidence interval; PCS, pathomics-collagen signature; LNM, lymph node metastasis; NRI, net reclassification improvement; IDI, integrated discrimination improvement.

# V. Supplementary References

1. Adomshick V, Pu Y, Veiga-Lopez A: Automated lipid droplet quantification system for phenotypic analysis of adipocytes using CellProfiler**.** *Toxicol Mech Methods* 2020, **30:**378-387.

2. Carpenter AE, Jones TR, Lamprecht MR, Clarke C, Kang IH, Friman O, Guertin DA, Chang JH, Lindquist RA, Moffat J, et al: CellProfiler: image analysis software for identifying and quantifying cell phenotypes**.** *Genome Biol* 2006, **7:**R100.

3. Logan DJ, Shan J, Bhatia SN, Carpenter AE: Quantifying co-cultured cell phenotypes in high-throughput using pixel-based classification**.** *Methods* 2016, **96:**6-11.

4. Weiss T, Semmler L, Millesi F, Mann A, Haertinger M, Salzmann M, Radtke C: Automated image analysis of stained cytospins to quantify Schwann cell purity and proliferation**.** *PLoS One* 2020, **15:**e0233647.

5. Ruifrok AC, Johnston DA: Quantification of histochemical staining by color deconvolution**.** *Anal Quant Cytol Histol* 2001, **23:**291-299.

6. Aaron JS, Taylor AB, Chew TL: Image co-localization - co-occurrence versus correlation**.** *J Cell Sci* 2018, **131:**jcs211847.

7. Haralick RM, Sternberg SR, Zhuang X: Image analysis using mathematical morphology**.** *IEEE Trans Pattern Anal Mach Intell* 1987, **9:**532-550.

8. Maragos PA: Pattern spectrum and multiscale shape representation. IEEE Trans Pattern Anal Mach Intell**.** *IEEE Transactions on Pattern Analysis and Machine Intelligence* 1989, **11:**701-716.

9. Vincent L: **Morphological area opening and closing for gray scale images.** In *Proceedings of NATO: Shape in picture workshop*. 1992: 197-208.

10. Vincent L: Granulometries and Opening Trees**.** *Fund Inform* 2000, **41:**57-90.

11. Wang G, Sun Y, Chen Y, Gao Q, Peng D, Lin H, Zhan Z, Liu Z, Zhuo S: Rapid identification of human ovarian cancer in second harmonic generation images using radiomics feature analyses and tree-based pipeline optimization tool**.** *J Biophotonics* 2020, **13:**e202000050.

12. Dempster AP: Maximum likelihood from incomplete data via the EM algorithm**.** *J R Stat Soc* 1977, **39:**1-38.

13. Stein AM, Vader DA, Jawerth LM, Weitz DA, Sander LM: An algorithm for extracting the network geometry of three-dimensional collagen gels**.** *J Microsc* 2010, **232:**463-475.

14. Frisch KE, Duenwald-Kuehl SE, Kobayashi H, Chamberlain CS, Lakes RS, Vanderby R, Jr.: Quantification of collagen organization using fractal dimensions and Fourier transforms**.** *Acta Histochem* 2012, **114:**140-144.

15. Haralick RM, Shanmugam K, Dinstein I: Textural Features for Image Classification**.** *Studies in Media and Communication* 1973, **SMC-3:**610-621.

16. Daugman JG: Complete discrete 2-D Gabor transforms by neural networks for image analysis and compression**.** *IEEE Transacoustspeech & Signal Process* 1988, **36:**1169-1179.

17. Jian H, Ma S, Zhang CH: Adaptive LASSO for sparse high-dimensional regression**.** *Stat Sin* 2008, **18:**1603-1618.

18. Meier L, Geer SVD, Bhlmann P, Zrich ETH: The group Lasso for logistic regression**.** *J R Stat Soc B* 2008, **70:**53-71.

19. Fitzgerald M, Saville B, Lewis R: Decision curve analysis**.** *JAMA* 2015, **313:**409-410.

20. Vickers AJ, Elkin EB: Decision Curve Analysis: A Novel Method for Evaluating Prediction Models**.** *Med Decis Making* 2006, **26:**565-574.

21. KF K, MD B, K Z, H J: Assessing the Clinical Impact of Risk Prediction Models With Decision Curves: Guidance for Correct Interpretation and Appropriate Use**.** *J Clin Oncol* 2016, **34:**2534-2540.

22. Bragg F, Trichia E, Aguilar-Ramirez D, Besevic J, Lewington S, Emberson J: Predictive value of circulating NMR metabolic biomarkers for type 2 diabetes risk in the UK Biobank study**.** *BMC Med* 2022, **20:**159.

23. Zhou N, Ji Z, Li F, Qiao B, Lin R, Jiang W, Zhu Y, Lin Y, Zhang K, Li S, et al: Machine Learning-Based Personalized Risk Prediction Model for Mortality of Patients Undergoing Mitral Valve Surgery: The PRIME Score**.** *Front Cardiovasc Med* 2022, **9:**866257.
